# Mechanisms for radical reactions initiating from *N*-hydroxyphthalimide esters

**DOI:** 10.3762/bjoc.20.35

**Published:** 2024-02-21

**Authors:** Carlos R Azpilcueta-Nicolas, Jean-Philip Lumb

**Affiliations:** 1 Department of Chemistry, McGill University, 801 Sherbrooke Street West, Montreal, Quebec H3A 0B8, Canada

**Keywords:** decarboxylative couplings, mechanisms, NHPI-esters, radical reactions

## Abstract

Due to their ease of preparation, stability, and diverse reactivity, *N*-hydroxyphthalimide (NHPI) esters have found many applications as radical precursors. Mechanistically, NHPI esters undergo a reductive decarboxylative fragmentation to provide a substrate radical capable of engaging in diverse transformations. Their reduction via single-electron transfer (SET) can occur under thermal, photochemical, or electrochemical conditions and can be influenced by a number of factors, including the nature of the electron donor, the use of Brønsted and Lewis acids, and the possibility of forming charge-transfer complexes. Such versatility creates many opportunities to influence the reaction conditions, providing a number of parameters with which to control reactivity. In this perspective, we provide an overview of the different mechanisms for radical reactions involving NHPI esters, with an emphasis on recent applications in radical additions, cyclizations and decarboxylative cross-coupling reactions. Within these reaction classes, we discuss the utility of the NHPI esters, with an eye towards their continued development in complexity-generating transformations.

## Introduction

The historical challenges of using radicals in synthetic chemistry is well documented [1–2]. Traditional approaches for radical generation relied on hazardous reagents and harsh conditions, resulting in low reaction efficiency and undesired byproduct formation [3–6]. As a consequence, the utility of radicals in organic synthesis remained limited for many years and in the past, they were perceived as fleeting reaction intermediates.

Recent progress in photoredox catalysis [6–8], electrochemistry [9–10], and the use of transition-metal (TM) catalysts in radical cross-coupling reactions [11] have dramatically expanded the use of radicals in synthesis, leading to their strategic incorporation as "synthons" in modern organic chemistry, with complementary reactivity to more common polar reaction manifolds [12–15]. The utility of radicals has also been expanded through the recent development of transformations involving radical-polar crossover, which incorporate both radical and ionic bond-forming steps into a single synthetic operation [16–17].

The success of radical reactions is intimately linked to the mechanisms of their initiation and the radical progenitor employed. Amongst the many progenitors that are available, carboxylic acids are one of the most extensively used, owing to their structural diversity and widespread commercial availability [18–19]. Carboxylic acids **1** can generate radicals under oxidative conditions, as in classical decarboxylative halogenation reactions (Hunsdiecker reaction) that proceed via a radical mechanism [20–21]. More recent approaches have leveraged photoinduced ligand-to-metal charge transfer to generate radicals from aliphatic [22] and aromatic [23–24] carboxylic acids.

**Scheme 1:**
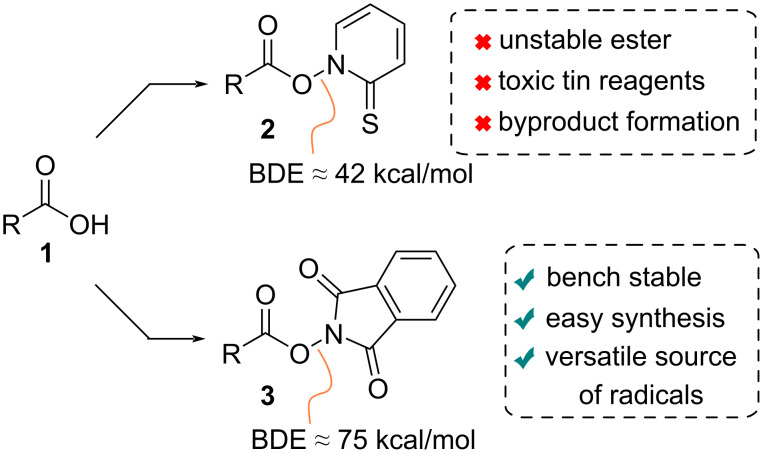
Comparison between Barton and NHPI ester radical precursors.

However, more broadly used approaches involve carefully designed activated esters. Barton esters **2** emerged in the early 1980s [25–26] (Scheme 1) and have found applications in a number of functional group interconversions mediated by radical chain decarboxylation [27]. However, their widespread use in synthesis, especially in complex molecular settings, suffers from significant disadvantages. These include thermal and photochemical instability, as evidenced by their low N–O bond dissociation energy (BDE ≈ 42 kcal/mol) [28], the reliance on toxic tin hydrides as reductants and the undesired radical recombination with reactive 2-pyridylthiyl radicals that leads to (alkylthio)pyridine byproducts [26]. More recently, *N*-hydroxyphthalimide (NHPI) esters (**3**) have emerged as convenient alternatives to Barton esters (Scheme 1) due in part to their ease of synthesis and greater stability (N–O BDE ≈ 75 kcal/mol) [28]. Their use as radical precursors was first described by Okada and colleagues in 1988 [29], who showed that C(sp^3^)-centered radicals were successfully generated by subjecting NHPI esters to light irradiation in the presence of the photoreductant 1,6-bis(dimethylamino)pyrene (BDMAP). Following their initial discovery, multiple studies have shown their versatility as radical progenitors under thermal, photochemical, and electrochemical conditions [30–31].

**Scheme 2:**
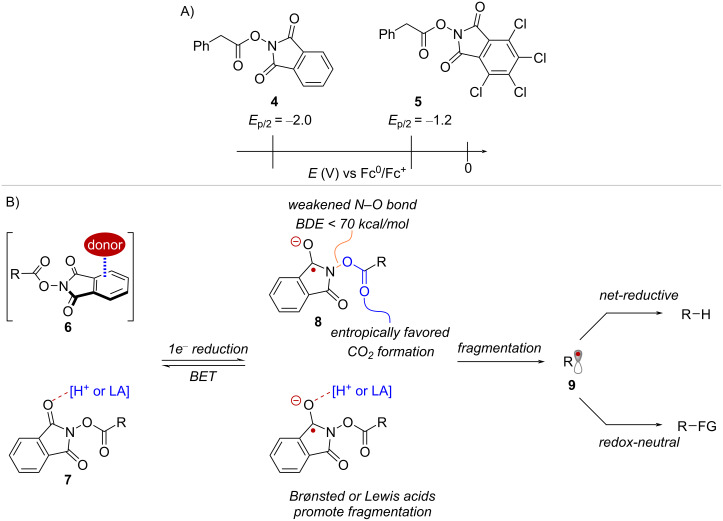
Overview of the mechanisms and activation modes involved in radical generation from RAEs.

Due to their propensity towards single-electron reduction, NHPI esters, and similar derivatives such as *N*-hydroxytetrachlorophthalimide (TCNHPI) esters, are collectively referred to as "redox-active esters" (RAEs). The versatility of RAEs stems, in part, to the sensitivity of their reduction half peak potentials (*E*_p/2_) to their environment. For instance, a recent study by Cornella and co-workers, showed that RAEs derived from phenylacetic acid exhibited varying reduction half peak potentials (*E*_p/2_) ranging from −2.0 V for the NHPI ester **4** to −1.2 V for the corresponding TCNHPI ester **5** (measured in MeCN vs Fc^0^/Fc^+^, see Scheme 2A) [32]. Based solely on their redox potentials, single-electron reduction of RAEs is only possible in the presence of a sufficiently strong reducing agent. However, the reduction of RAEs can also be facilitated through the formation of charge transfer complexes with a donor species **6** or via LUMO lowering activation with Brønsted and Lewis acids **7** (Scheme 2B), collectively offering a number of variables to influence their reactivity.

Upon reduction, RAEs give rise to a radical anion **8** with a weakened N–O bond (BDE < 70 kcal/mol) [33]. While fragmentation of **8** affords a radical species **9** in a constructive step towards initiating the radical reaction, a principal competing step is back-electron transfer (BET) to return the closed shell starting materials (Scheme 2B). A recent study showed comparable rates for fragmentation (8 ± 5 × 10^5^ s^−1^) and BET (3.15 to 2.06 × 10^5^ s^−1^) [34] when employing catalytic Ir^III^ excited state reductants with moderate reducing potentials (*E*_1/2_^red^[Ir^IV^/*Ir^III^] ≈ −1.13 V vs Fc^0^/Fc^+^ in MeCN) [35]. This suggests that in the absence of a sufficiently strong driving force, BET and fragmentation compete to influence the resulting concentration of radicals. In such instances, opting for a stronger catalytic reductant or utilizing a stoichiometric electron donor can greatly improve the efficiency of radical generation. On the other hand, additional factors such as the ability of Brønsted and Lewis acid additives to promote the fragmentation step can be considered. Importantly, the diverse mechanisms in which RAEs engage in radical reactions can be exploited to modulate the reactivity of the resulting substrate radicals. For example, under net-reductive conditions, the radical intermediates are typically terminated via hydrogen atom transfer (HAT) or sequential electron transfer and proton transfer (ET/PT) steps. Alternatively, redox-neutral transformations can be envisioned using catalytic reductants, which can enable a complementary scope of downstream functionalizations (Scheme 2B).

In this perspective, we present an overview of the diverse mechanisms that have been proposed for radical based transformations initiating from NHPI esters. The discussion is organized into four sections: (i) mechanisms under photochemical conditions, (ii) initiation by metal catalysis and stoichiometric reductants, (iii) *N*-heterocyclic carbene (NHC)-catalyzed radical relay, and (iv) mechanisms under electrochemical activation.

By discussing selected literature examples, we illustrate how the activation mode of NHPI esters, and the reactivity of the resulting radical species, can vary depending upon the choice of catalytic or stoichiometric electron donors, the presence of a TM catalyst, the formation of a charge-transfer complex, and the overall reaction conditions. While we hope that this discussion will spur the continued development of NHPI esters in complexity-generating transformations, it is not comprehensive, and we refer readers to recently published review articles for additional discussion [30–31].

## Discussion

### Mechanism under photochemical conditions

**Scheme 3:**
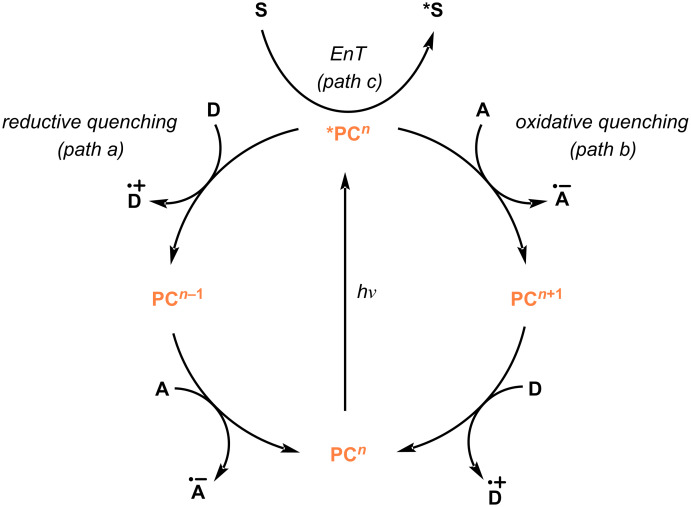
Common mechanisms in photocatalysis.

In this section we provide a summary of the various conditions and activation modes employed in radical reactions of NHPI esters using visible-light irradiation. Upon absorption of light, an excited photocatalyst (*PC) engages in single-electron transfer (SET) with either donor (**D**) or acceptor (**A**) molecules (Scheme 3) [8,36]. Accordingly, a reductive quenching mechanism (path a) will operate when an excited photocatalyst effects the one-electron oxidation of a sacrificial donor giving rise to a strongly reducing catalytic species (**PC*^n^*^−1^**). On the other hand, in an oxidative quenching mechanism (path b) the excited photocatalyst directly induces the one-electron reduction of an acceptor substrate. Alternatively, the photocatalyst can mediate the formation of an electronically excited substrate (*S) through an energy transfer (EnT) mechanism (path c).

In addition to these mechanistic blueprints, the formation of charge-transfer complexes involving NHPI esters, as well as examples of photoinduced transition metal-catalyzed activation will be discussed. Depending on the specific activation mechanism, both net reductive and redox neutral transformations can be implemented.

### Photocatalytic reductive quenching mechanism

**Scheme 4:**
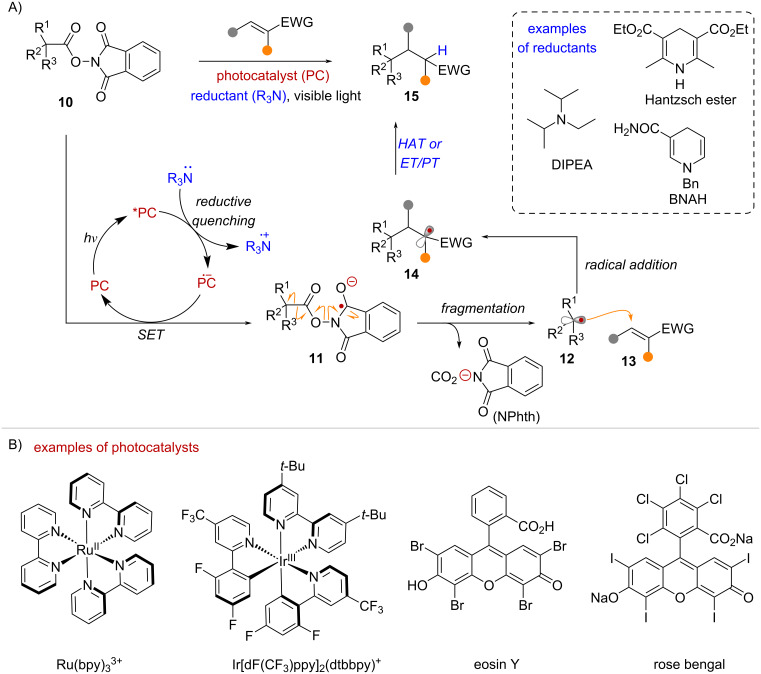
A) Giese-type radical addition of NHPI esters mediated by a reductive quenching photocatalytic cycle. B) Examples of common photocatalysts.

Among the most common reactions of NHPI esters are radical additions to electron-deficient olefins under net-reductive conditions, often referred to as Giese type addition reactions (Scheme 4A). In 1991, Okada and co-workers reported the addition of alkyl radicals to α,β-unsaturated ketones, by subjecting NHPI esters to visible-light irradiation in the presence of the photocatalyst [Ru(bpy)_3_]Cl_2_ and the reductant 1-benzyl-1,4-dihydronicotinamide (BNAH) [37]. Two decades later, in 2012 the Overman group demonstrated the utility of this transformation in the total synthesis of (–)-aplyviolene, involving the diastereoselective coupling of a tertiary radical and an enone acceptor [38]. Further developments of this chemistry resulted in the general use of NHPI esters for the construction of quaternary carbons via conjugate addition of 3° radicals [39–40]. In general, this transformation operates under a reductive quenching photocatalytic cycle, requiring a stoichiometric reductant (Scheme 4A). Both TM complexes, and organic dyes such as eosin Y [41–43], have been employed as suitable photocatalysts (Scheme 4B). Under visible light irradiation the photocatalyst (**PC**) is excited into its corresponding excited state (*PC), where it can be reduced by a suitable electron donor such as DIPEA or Hantzsch ester to generate the reduced form of the photocatalyst (**PC^•–^**) (Scheme 4A). This strong reducing agent mediates the one-electron reduction of the NHPI ester **10**, forming radical anion intermediate **11**. Fragmentation of **11** via N–O bond homolysis and decarboxylation forms the key tertiary radical **12** with concomitant formation of phthalimidyl anion (**^–^**Nphth) and CO_2_. Radical **12** undergoes intermolecular addition to the olefin acceptor **13** to form radical intermediate **14**. Finally, under reductive conditions radical **14** can undergo hydrogen atom transfer (HAT) or sequential electron transfer and proton transfer (ET/PT) to form the conjugate addition product **15**.

**Scheme 5:**
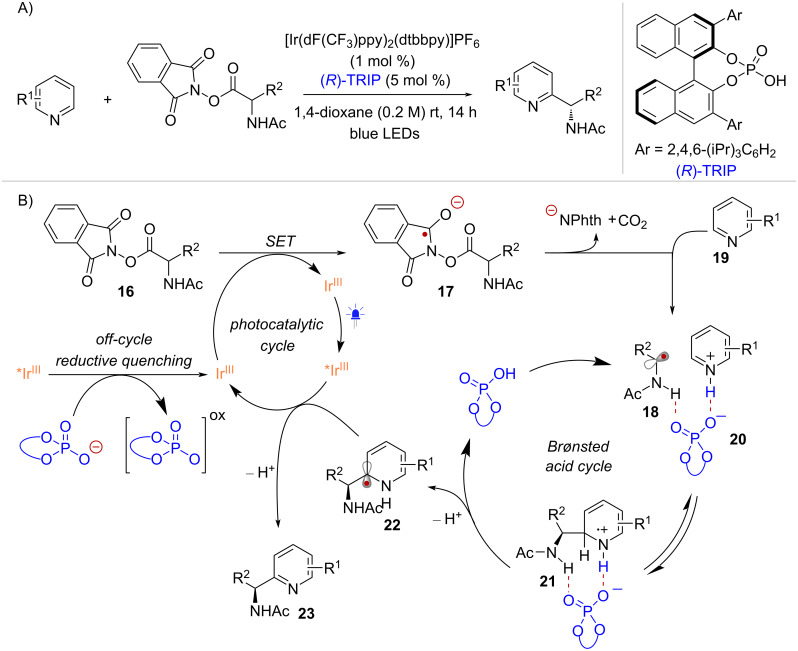
A) Minisci-type radical addition of NHPI esters. B) Reaction mechanism involving an “off-cycle” reductive quenching.

With this mechanistic blueprint as a backdrop, Phipps and co-workers developed an enantioselective Minisci-type addition, under dual photoredox and chiral Brønsted acid catalysis [44] (Scheme 5A). In their proposed mechanism, the activation of the NHPI ester radical precursor was proposed to occur via a reductive quenching mechanism. However, since the overall transformation is redox-neutral, no stoichiometric reductant was employed. Instead, fluorescence-quenching studies suggested that the reductive quenching of the iridium excited state (*Ir^III^) was taking place "off-cycle" via oxidation of the chiral phosphate co-catalyst (Scheme 5B). This event leads to the generation of a potent Ir^II^ reductant that begins the photocatalytic cycle by reducing **16** into radical anion **17** while regenerating the ground state of the Ir^III^ photocatalyst. After fragmentation, α-amino radical **18** was proposed to undergo addition to the heterocyclic radical acceptor **19** through a ternary transition state **20** involving hydrogen bonding interactions with the chiral phosphate co-catalyst. Notably, a follow-up report revealed that the radical addition is reversible, and that the selectivity determining step involves the deprotonation of **21** to provide radical intermediate **22** [45]. Finally, the iridium excited state (*Ir^III^) formed under blue light irradiation oxidizes **22** to form product **23** and the corresponding reduced Ir^II^ complex, beginning a new photocatalytic cycle.

### Photocatalytic oxidative quenching mechanism

**Scheme 6:**
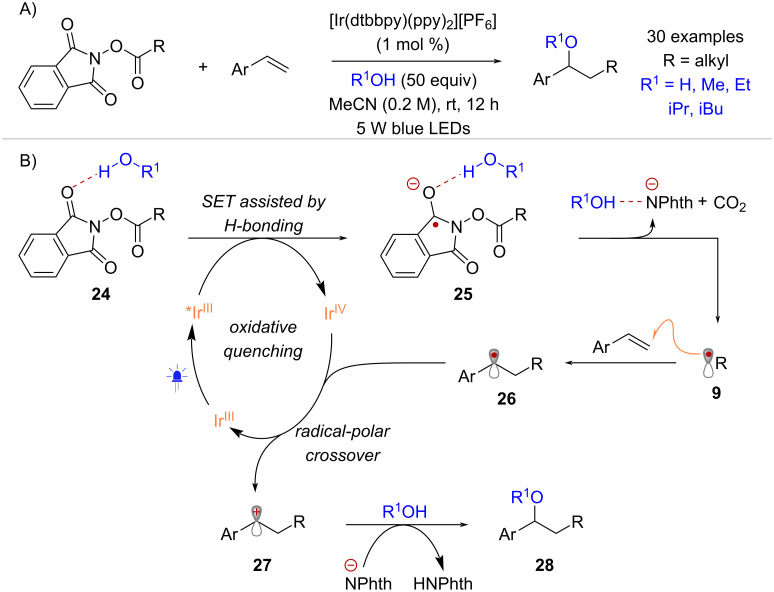
Activation of NHPI esters through hydrogen-bonding in an oxidative quenching photocatalytic cycle.

The activation of NHPI esters under a photocatalytic oxidative quenching mechanism was reported for the first time by Glorius and co-workers in 2017 [46]. This activation mode was applied in the functionalization of styrenes using an Ir-photocatalyst and a diverse range of nucleophiles that are H-bond donors (Scheme 6A). Stern–Volmer analysis revealed that quenching of the photocatalyst’s excited state by the NHPI ester occurred only in presence of a hydrogen bond donor such as water (H_2_O) or methanol (MeOH). This supported the hypothesis that activation of NHPI esters towards photoinduced electron transfer can occur through hydrogen bonding. In the proposed mechanism (Scheme 6B), hydrogen bonded complex **24** undergoes single electron reduction via oxidative quenching of the excited state *Ir^III^, resulting in the formation of radical anion **25** (presumably H-bonded to H_2_O) and the corresponding Ir^IV^ complex. Radical intermediate **9** formed upon fragmentation of **25**, adds to the styrene acceptor forming radical **26**. Finally, a radical-polar crossover event between **26** and the Ir^IV^ complex regenerates the Ir^III^ ground state while delivering cation **27** that is then trapped by the oxygen-nucleophile to form the oxyalkylation product **28**.

**Scheme 7:**
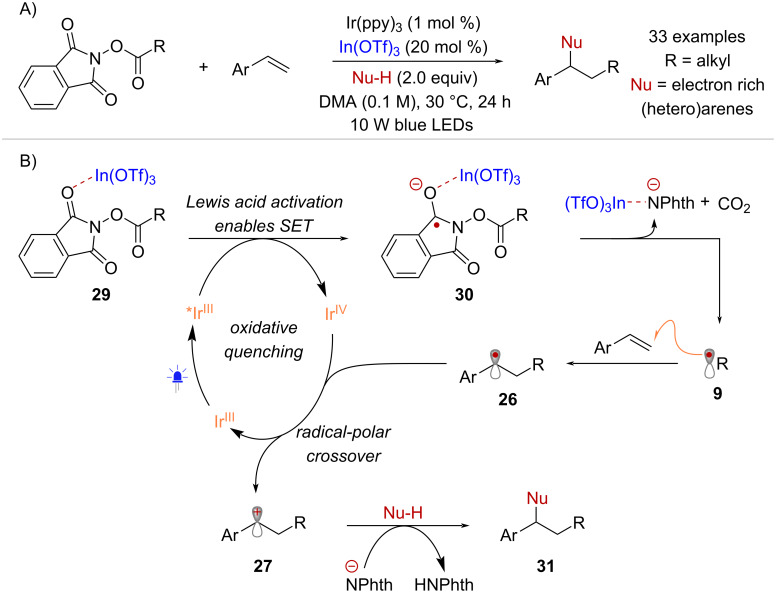
SET activation of RAE facilitated by a Lewis acid catalyst.

Li and co-workers described the activation of NHPI esters towards SET using a Lewis acid catalyst, allowing for the functionalization of styrene radical acceptors with nucleophiles that do not necessarily engage in hydrogen-bonding interactions, such as electron-rich (hetero)arenes [47] (Scheme 7A). Cyclic voltammetry measurements of a model NHPI ester showed a shift in its reduction potential from –1.79 V to –1.51 V (vs SCE in MeCN) in the presence of In(OTf)_3_. As such, it was hypothesized that the Lewis acid lowers the LUMO of the NHPI ester via interaction with the oxygen lone pair in the phthalimide moiety (Scheme 7B). Thus, the excited state reductant *Ir^III^ reduces the activated substrate **29** to form the stabilized radical anion **30**. Fragmentation into radical **9**, followed by radical addition to styrene gives benzyl radical intermediate **26**. Turn-over of the catalytic cycle through radical-polar crossover affords cation **27** that delivers functionalized product **31** upon nucleophilic addition.

**Scheme 8:**
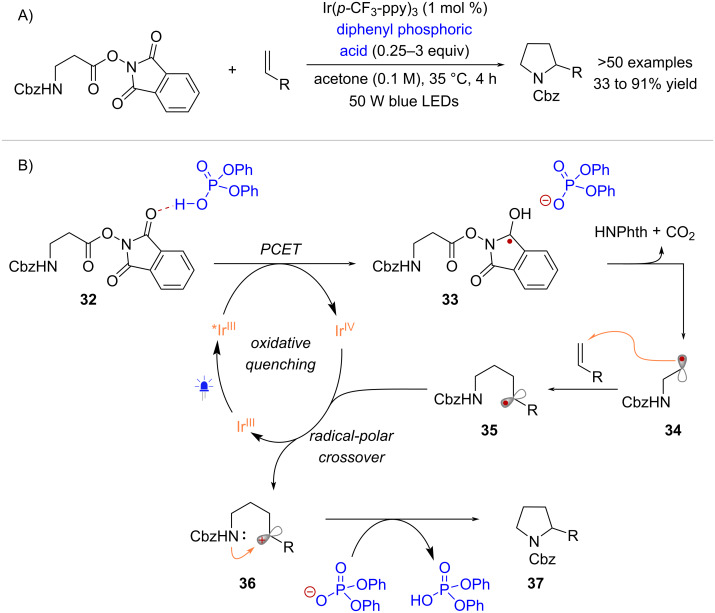
PCET activation of NHPI esters in the context of a radical-redox annulation.

The Doyle and Knowles groups reported the use of NHPI esters as radical precursors in the context of a radical redox annulation method [48] (Scheme 8A). This transformation occurs through an oxidative quenching photocatalytic cycle employing Ir-based photoreductants and a Brønsted acid additive. While the interaction between the RAE **32** and diphenyl phosphoric acid involves hydrogen bonding, in analogy to the Glorius proposal, it is thought that the substrate activation occurs through proton-coupled electron transfer (PCET), forming neutral radical species **33** and the corresponding phosphate conjugate base (Scheme 8B). The hypothesis is supported by an observed increase in the luminescence quenching of *Ir(p-CF_3_-ppy)_3_ by **32** in the presence of diphenyl phosphoric acid, as quantified by the Stern–Volmer constant (*K*_sv_ = 1146 M^−1^ with acid vs *K*_sv_ = 603 M^−1^ without acid). The reaction mechanism continues with the fragmentation of **33** into radical **34**. From radical **34** the annulation reaction initiates via intermolecular radical addition, resulting in the formation of intermediate **35**. After oxidation of **35** to **36**, the photocatalyst is regenerated and product **37** is formed through intramolecular nucleophilic cyclization facilitated by the phosphate base. Importantly, the activation of NHPI esters through PCET may also play a role in transformations mediated by the cyanoarene-based donor–acceptor photocatalyst 4CzIPN, which typically requires the use of strong H^+^ donors such as trifluoroacetic acid [49–52]. While many reports, including Okada’s original work, have suggested that a proton donor can accelerate the fragmentation rate of RAEs [29,49,53], specific values comparing the fragmentation rate constants or the N–O bond dissociation energies of radical anions and neutral radicals derived from RAEs are not available in the literature.

**Scheme 9:**
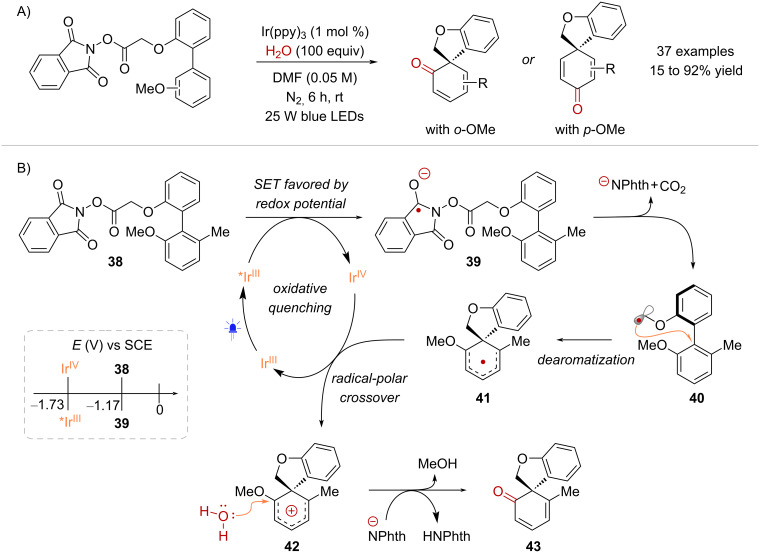
Activation enabled by a strong excited-state reductant catalyst and its application in the dearomative spirocyclization of biaryls.

Lumb and co-workers recently published a study on the conversion of biaryl-derived NHPI esters into spirocyclic cyclohexadienones through a photocatalytic radical-mediated dearomatization, with H_2_O serving as the nucleophile [54] (Scheme 9A). Despite the presence of H_2_O in the reaction, the reduction of **38** to its corresponding radical anion **39** could occur without the need for hydrogen-bonding (Scheme 9B). Cyclic voltammetry measurements of NHPI ester **38** displayed a reduction half peak potential (*E*_p/2_) of −1.17 V (vs SCE in MeCN) indicating that the single-electron reduction of **38** by a suitably strong reductant, such as *Ir(ppy)_3_ (*E*_1/2_^red^[IrIV/*IrIII] = −1.73 V vs SCE in MeCN) would be thermodynamically favorable. Indeed, Stern–Volmer photoquenching experiments confirmed that **38** effectively quenched *Ir(ppy)_3_ under anhydrous conditions. Consequently, the SET reduction of **38**, followed by fragmentation of **39** yielded α-oxy radical intermediate **40**. Subsequently, the spirocyclization of **40** induced the dearomatization of the methoxy-substituted aromatic ring, forming intermediate **41**, which was then oxidized to cation **42**, thereby completing the photocatalytic cycle. The reaction proceeded by regioselective nucleophilic addition of H_2_O, accompanied by the loss of MeOH to deliver spirocycle **43**. Notably, the dearomative spirocyclization of biaryl-derived NHPI esters has found application in the total synthesis of natural products, including the plant metabolite denobilone A and the highly oxidized dibenzocyclooctadiene lignans heteroclitin J and kadsulignan E [55].

### Activation via charge-transfer complex formation

**Scheme 10:**
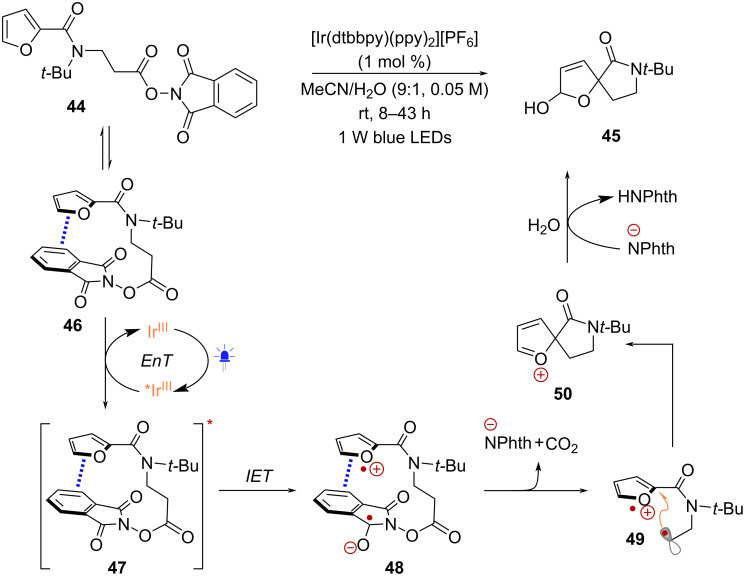
Proposed formation of an intramolecular charge-transfer complex in the synthesis of (spiro)anellated furans.

Under conditions where oxidative quenching is not thermodynamically favorable, the single electron reduction of RAEs can proceed via an alternative mechanism. For example, in the transformation of NHPI ester **44** into spirocycle **45** catalyzed by [Ir(ppy)_2_(dtbbpy)][PF]_6_, Reiser and co-workers observed that the excited state of this Ir-catalyst (*E*_1/2_^red^[Ir^IV^/*Ir^III^] = −0.96 V vs SCE) would not promote the reduction of **44** (−1.25 V vs SCE) [56] (Scheme 10). Interestingly, the role of H-bonding in substrate activation was not considered. To explain the observed transformation, it was suggested that the Ir^III^-photocatalyst acted as a photosensitizer in an energy-transfer (EnT) mechanism. This proposal was supported by fluorescence quenching measurements, as well as the direct excitation of **44** by UV irradiation, resulting in the formation of **45** in a 45% yield. According to this hypothesis, NHPI ester **44** would adopt a favorable conformation (**46**) for π-stacking between the furan and phthalimide rings, before EnT from *Ir^III^ leads to the formation of an excited charge-transfer complex **47**. This species would undergo intramolecular electron transfer (IET) giving rise to intermediate **48**, which upon fragmentation would form radical **49**. Intramolecular radical addition into the radical cation of the furan ring would then form cation **50** before nucleophilic capture by H_2_O leads to product **45**.

**Scheme 11:**
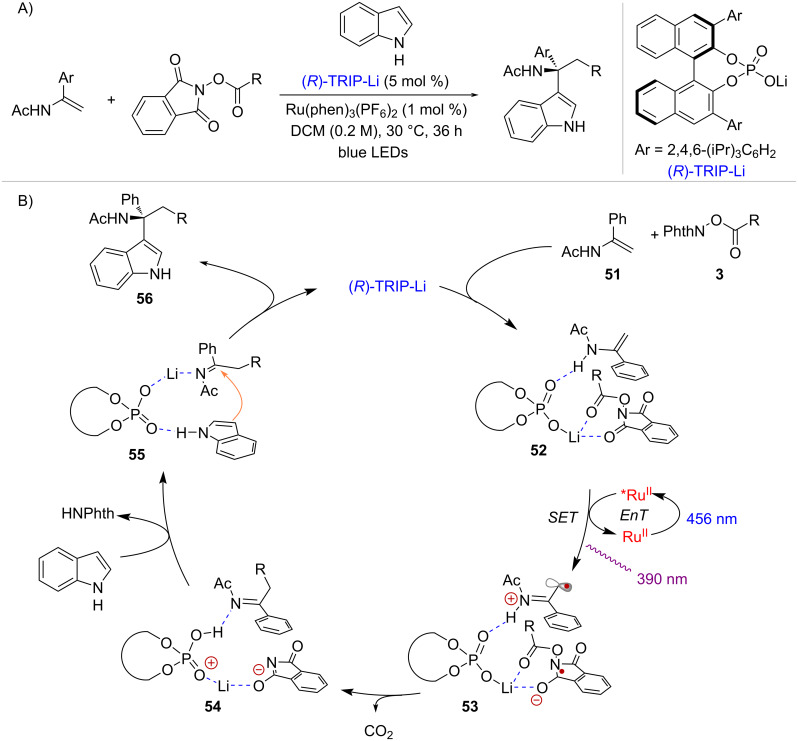
Formation of a charge-transfer complex between enamides and NHPI esters enabled by a chiral phosphate catalyst.

In 2020, the Wang group reported the functionalization of enamides employing radicals derived from NHPI esters in combination with indole nucleophiles [57] (Scheme 11A). This transformation occurred under light irradiation either in the presence or absence of a Ru^II^ photoredox catalyst. It was found that the chiral lithium phosphate catalyst (*R*)-TRIP-Li played a crucial role in accelerating the reaction rate. Following an in-depth analysis of the mechanism, the authors proposed that (*R*)-TRIP-Li has the capability to engage enamide **51** through H-bonding and NHPI ester **3** through Li-promoted Lewis acid activation, acting as a pocket that facilitates the formation of charge-transfer complex **52** (Scheme 11B). This complex can be excited either by direct irradiation at 390 nm or through Ru^II^-mediated EnT under blue light irradiation (456 nm). Following excitation, SET from the enamide to the active ester forms intermediate **53**, which undergoes fragmentation and radical recombination to afford intermediate **54**. At this stage, the indole nucleophile substitutes the phthalimidyl anion within the chiral pocket of the phosphate catalyst to form complex **55**, before enantioselective addition to the iminium ion affords product **56**.

**Scheme 12:**
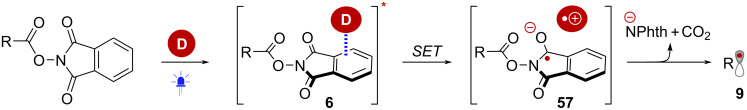
Activation of NHPI ester through the formation of photoactive EDA-complexes.

NHPI esters can also engage in π–π interactions with electron-rich species to generate charge-transfer complexes that can absorb light in the visible region. These species are referred to in the literature as electron donor–acceptor (EDA) complexes [58–59] and undergo photoexcitation in the absence of an exogenous photoredox catalyst. When excited by visible light, an intra-complex SET from the donor substrate **D** to the NHPI ester acceptor takes place, generating radical ion pair **57**. Subsequently, this ion pair undergoes fragmentation, forming the corresponding substrate radical **9**, which can participate in diverse chemical transformations (Scheme 12).

**Scheme 13:**
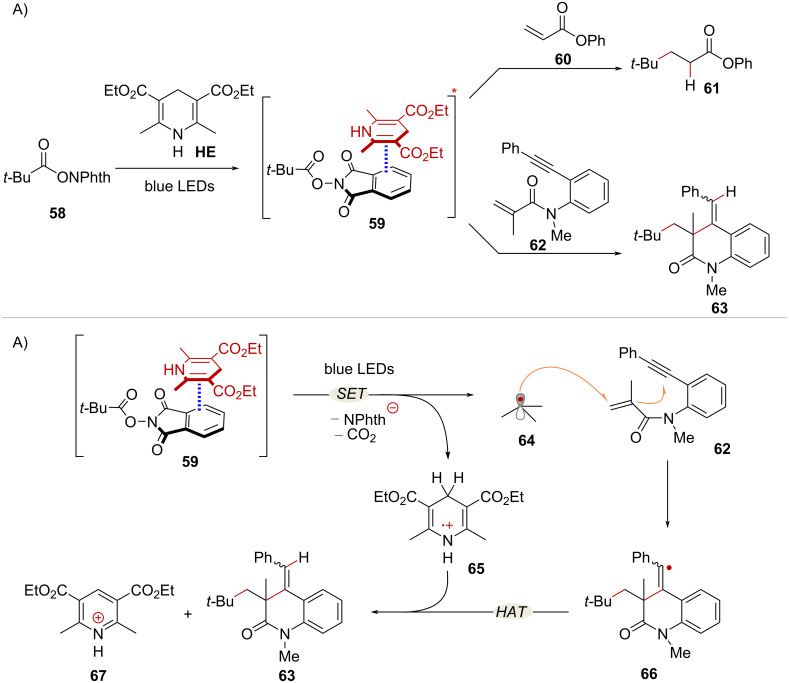
A) EDA complex-mediated radical hydroalkylation reactions of NHPI esters. B) Proposed mechanism for the hydroalkylation cascade.

Different donor molecules are known to form EDA complexes with NHPI esters. For example, the NHPI ester derived from pivalic acid **58** and Hantzsch ester **HE** form EDA complex **59** which participates in radical mediated hydroalkylation reactions [60–61] (Scheme 13A). In the presence of electron deficient olefin **60**, classic Giese-type addition takes place under photocatalyst-free conditions, affording product **61** [60]. On the other hand, reaction with 1,7-enyne **62** affords dihydroquinolinone product **63** via a cascade radical addition/cyclization process [61]. In both transformations, **HE** serves a dual role by activating the NHPI ester through EDA complex formation and providing a hydrogen atom to terminate the radical reaction. The proposed mechanism of the hydroalkylation cascade is depicted in Scheme 13B. Upon excitation of complex **59** with blue light, intra-complex SET takes place from the **HE** to the NHPI ester, leading to the formation of *tert*-butyl radical **64** and radical cation **65**. Addition of radical **64** to enyne **62** followed by *6-exo-dig* cyclization yields radical intermediate **66**. Finally, species **65** acts as a hydrogen atom donor, delivering product **63** while forming pyridine **67** as a byproduct.

**Scheme 14:**
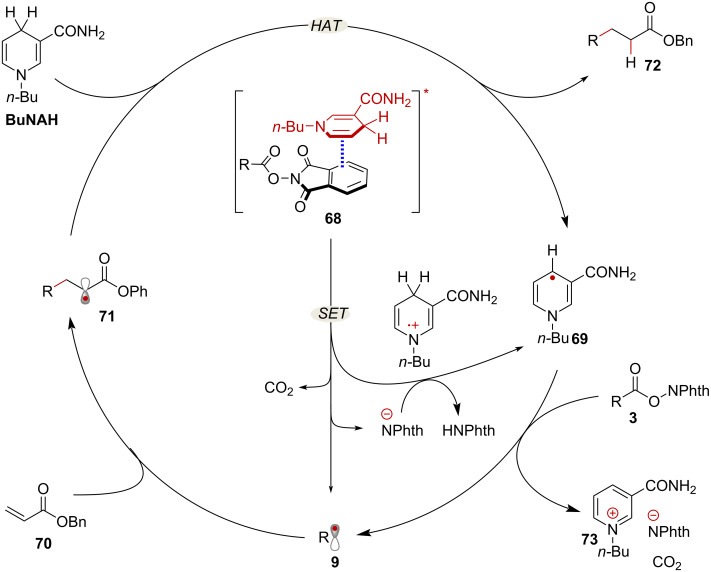
Proposed radical chain mechanism initiated by EDA-complex formation.

A similar mechanism would in principle account for EDA-complex-mediated Giese-type additions. However, an alternative radical chain mechanism has been discussed in the literature [62] (Scheme 14). In this instance, chain initiation takes place through photoinduced SET, enabled by EDA complex formation between the reductant *N*-(*n*-butyl)-1,4-dihydronicotinamide (**BuNAH**) and an NHPI ester (complex **68**). This process delivers substrate radical **9** and nicotinyl radical **69** following proton transfer to the phthalimidyl anion. Then, addition of **9** to α,β-unsaturated ester **70** yields radical intermediate **71**. At this stage, HAT mediated by another equivalent of **BuNAH** delivers product **72**, with concomitant formation of radical **69**. Finally, aromatization of **69** via SET to NHPI ester **3**, generates pyridinium **73** as a byproduct, while propagating the radical chain reaction.

**Scheme 15:**
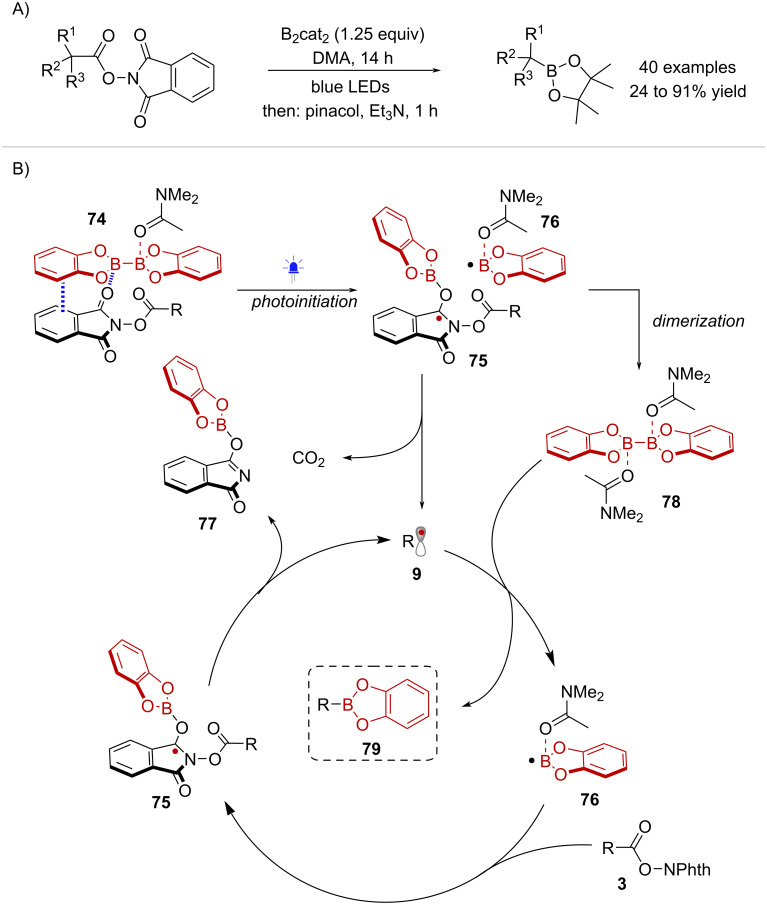
A) Photoinduced decarboxylative borylation. B) Proposed radical chain mechanism.

Aggarwal and co-workers discovered the photoinduced decarboxylative borylation of NHPI esters mediated by bis(catecholato)diboron (B_2_cat_2_) [63] (Scheme 15A). UV–vis absorption measurements showed that a mixture of a model NHPI ester with B_2_cat_2_ in dimethylacetamide (DMA) formed a new charge-transfer band in the visible region (>390 nm), which was attributed to the formation of EDA complex **74** (Scheme 15B). Under blue light irradiation, EDA complex **74** triggered a radical chain process initiated by B–B bond cleavage, forming boryl-NHPI ester radical **75** and boryl radical **76**. Subsequent decarboxylation of **75** yields carbon-centered radical **9** and boryl-phthalimide byproduct **77**. Meanwhile DMA-ligated B_2_cat_2 _**78** is formed upon dimerization of radical **76**. Reaction between radical **9** and species **78** affords boronic ester **79** while returning boryl radical **76**. Finally, chain propagation takes place via reaction of **76** with another equivalent of the NHPI ester **3**.

The Baran lab has recently published a complementary electrochemical method, wherein the activation of complex **74** takes place through SET under constant current electrolysis [64]. The Glorius and Y. Fu groups have independently proposed the formation of analogous charge-transfer complexes involving NHPI esters, bis(pinacolato)diboron (B_2_pin_2_), and Lewis bases (pyridine or isonicotinate *tert*-butyl ester) in C(sp^2^)-borylation methods under photochemical and thermal conditions, respectively [65–66].

**Scheme 16:**
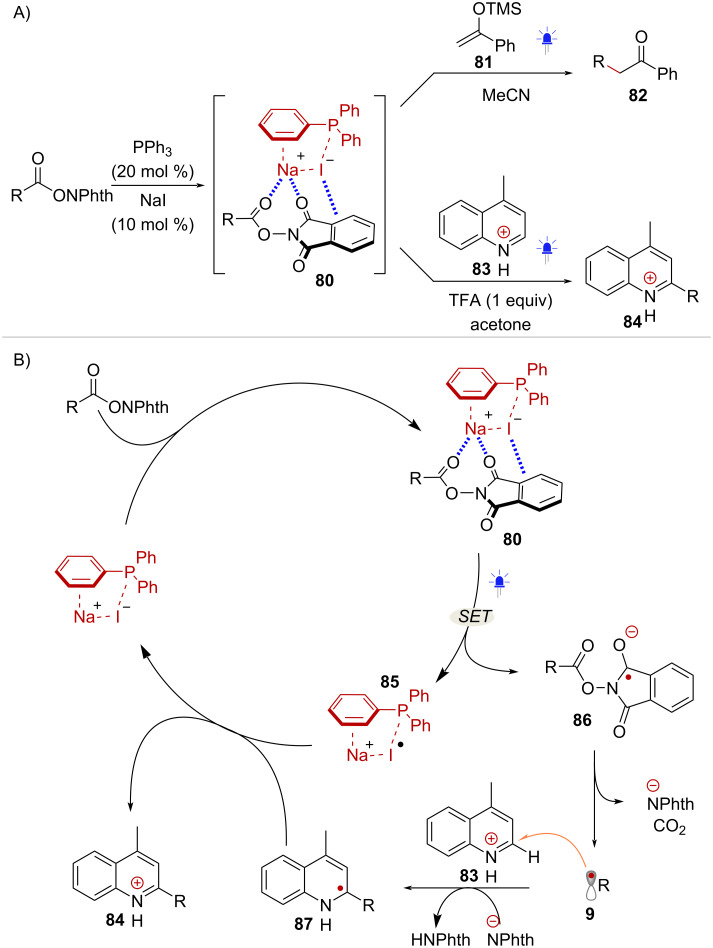
A) Activation of NHPI esters mediated by PPh_3_/NaI. B) Proposed catalytic cycle involving EDA-complex formation.

The activation of NHPI esters through EDA complex formation is also possible by employing a catalytic donor species, which enables a range of redox neutral transformations. In 2019, Shang and Fu initially demonstrated this approach by utilizing catalytic amounts of triphenylphosphine (PPh_3_) and sodium iodide (NaI) [67]. Upon formation of EDA complex **80**, radical addition to silyl enol ether **81** was promoted under blue light irradiation, affording acetophenone product **82** (Scheme 16A). Additionally, Minisci-type additions were carried out in the presence of protonated quinoline radical acceptor **83**, affording product **84** (Scheme 16A). Mechanistically, this activation mode involves an intra-complex SET that forms the Ph_3_P–NaI radical cation species **85** and the corresponding radical anion **86**. Decarboxylative fragmentation of **86** forms radical **9**, which upon radical addition to **84** and deprotonation yields radical **87**. Finally, oxidation of **87** mediated by **85** delivers the Minisci addition product **84** while regenerating PPh_3_ and NaI (Scheme 16B).

**Scheme 17:**
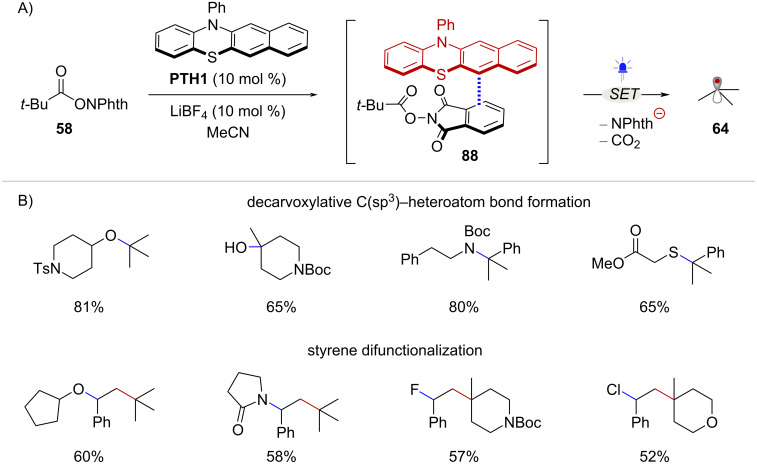
A) Radical generation facilitated by EDA complex formation between **PTH1** catalyst and NHPI esters. B) Selected scope of **PTH1**-catalyzed decarboxylative transformations.

The Ohmiya group has developed a series of light-mediated decarboxylative transformations of NHPI esters using a phenothiazine-based organophotoredox catalyst **PTH1**. This type of catalyst is believed to facilitate SET to NHPI esters through the formation of EDA complexes. Interestingly, upon addition of RAE **58** to a solution of **PTH1**, a noticeable red shift in the UV–vis absorption spectra of **PTH1** was observed, suggesting the formation of a charge transfer complex of the type **88** [68] (Scheme 17A). *tert*-Butyl radical (**64**), along with additional 2° and 3° alkyl radicals, resulting from these EDA complexes were harnessed in C(sp^3^)-heteroatom bond forming reactions [69], and in the difunctionalization of styrenes [68] (Scheme 17B).

**Scheme 18:**
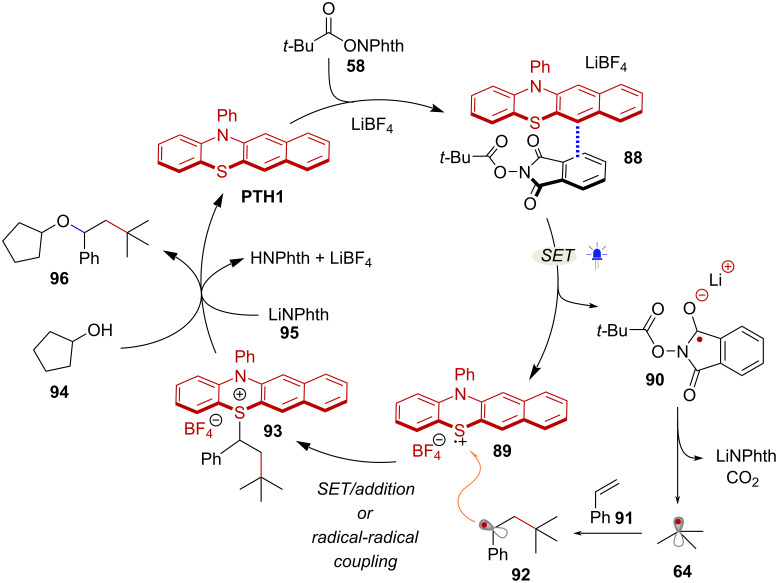
Proposed catalytic cycle for the difunctionalization of styrenes.

The catalytic cycle for the styrene difunctionalization reaction is depicted in Scheme 18. First, EDA complex **88** consisting of RAE **58** and the **PTH1** catalyst is formed. Under blue light irradiation, intra-complex SET leads to the formation of PTH radical cation species **89** and the radical anion **90** with the corresponding charges balanced by the LiBF_4_ additive. Notably, it was suggested that LiBF_4_ could also facilitate the reduction of the NHPI ester substrate, potentially by the coordination of Li cation to the phthalimide moiety. Next, fragmentation of **90** yields *tert*-butyl radical **64**, which then adds to styrene **91** affording radical intermediate **92**. At this stage, recombination of intermediates **89** and **92** may occur via SET followed by addition or through radical–radical coupling, affording benzylsulfonium intermediate **93**. Finally, nucleophilic substitution with alcohol **94** in the presence of lithium phthalimide **95** leads to product **96** and turns over the catalytic cycle. Importantly, species **93** can be detected by high resolution mass spectrometry, when the reaction is carried out without nucleophile and using stoichiometric amounts of **PTH1**.

**Scheme 19:**
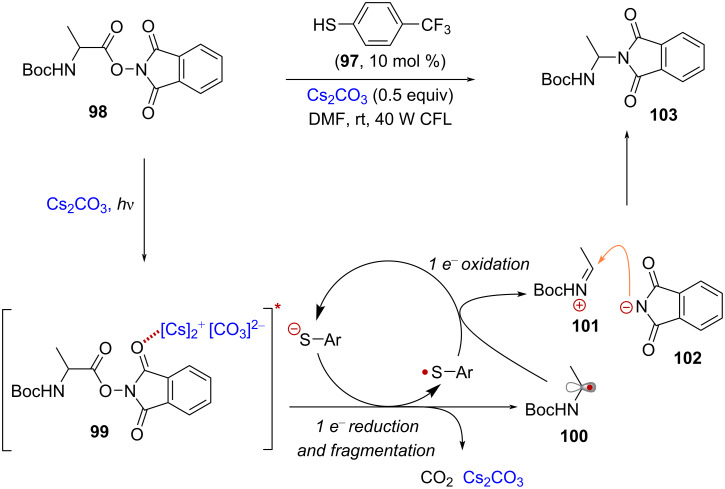
Formation of a charge-transfer complex between NHPI esters and Cs_2_CO_3_ enables decarboxylative amination.

H. Fu and co-workers reported that the combination of NHPI esters and cesium carbonate (Cs_2_CO_3_) gave rise to a new absorption band in the visible region, suggesting the formation of a photoactive charge-transfer complex [70]. This activation mode was initially employed in a decarboxylative coupling reaction with aryl thiols [70]. Further studies showed that 4-(trifluoromethyl)thiophenol (**97**) could act as a catalytic reductant in the aminodecarboxylation reaction of NHPI esters derived from α-amino acids [71] (Scheme 19). Accordingly, visible light irradiation of a mixture consisting of *N*-Boc-alanine NHPI ester **98** and Cs_2_CO_3_ in DMF resulted in the generation of the excited charge transfer complex **99**. Subsequent SET mediated by the thiol catalyst followed by fragmentation afforded α-amino radical **100**, which was then oxidized by the resulting thiol-radical species, regenerating the thiol catalyst while forming an iminium intermediate **101**. Finally, nucleophilic addition of phthalimide anion **102** afforded the amination product **103**.

**Scheme 20:**
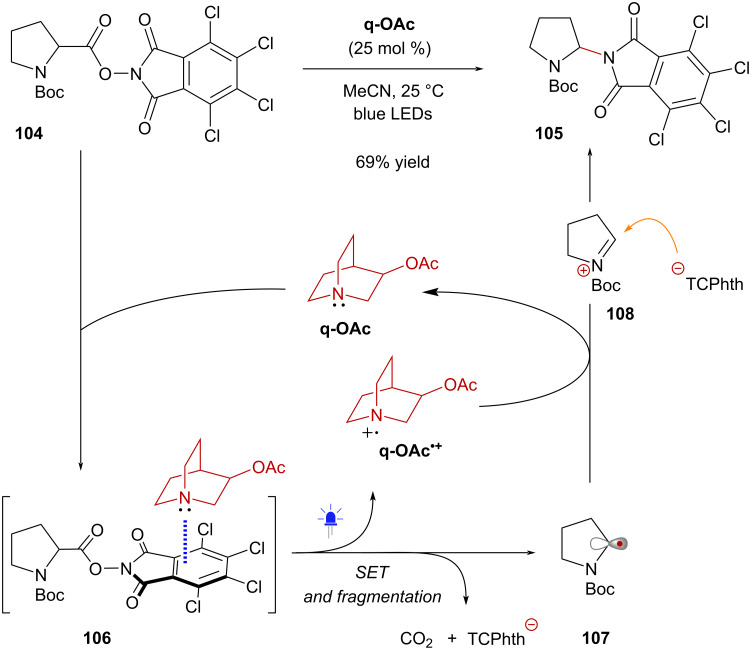
3-Acetoxyquinuclidine as catalytic donor in the activation of TCNHPI esters.

Bosque and Bach reported the use of 3-acetoxyquinuclidine (**q-Ac**) as a catalytic donor for the activation of TCNHPI esters derived from α-amino acids [72] (Scheme 20). Treatment of *N*-Boc-proline-derived TCNHPI ester **104** with **q-OAc** in MeCN resulted in the formation of a yellow solution, which upon blue light irradiation provided the aminodecarboxylation product **105** in 69% yield. It was hypothesized that the reaction involved the formation of EDA complex **106** that led to the formation of α-amino radical **107** through photoinduced SET followed by fragmentation. Subsequent oxidation of **107** by radical cation **q-Ac^•+^** afforded iminium ion **108** before nucleophilic addition of the in situ-generated tetrachlorophthalimyl anion (^–^TCPhth) led to the formation of aminal product **105**. Of note, the aminodecarboxylation reaction proved unsuccessful when employing alternative photocatalysts such as Ru(bpy)_3_Cl_2_ or eosin Y, underscoring the distinctive ability of **q-OAc** to activate TCNHPI esters via EDA complex formation.

### Photoinduced transition metal-catalyzed mechanisms

The in situ formation of photoactive catalysts can be achieved by combining simple transition metal (TM) salts with suitable ligands. These TM catalysts are fundamentally distinct from traditional Ru- and Ir-based photoredox catalysts, as they play a dual role, by engaging in photoinduced electron transfer processes with the substrate and participating in the key bond-forming/breaking steps via substrate–TM interactions [73–74]. This paradigm has been employed in the activation of NHPI esters under photoinduced copper (Cu) and palladium (Pd) catalysis.

**Scheme 21:**
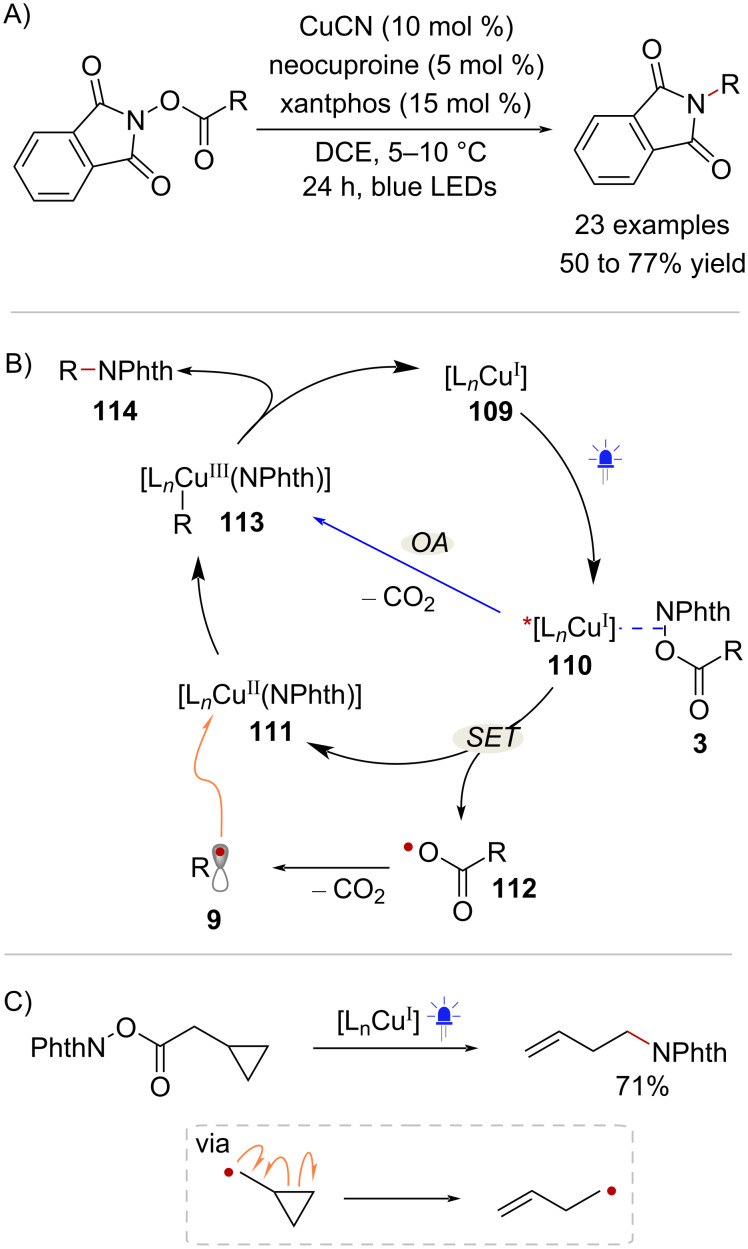
A) Photoinduced Cu-catalyzed decarboxylative amination. B) Proposed catalytic cycle. C) Radical clock experiment supporting the intermediacy of radicals.

In 2017, Peters and Fu reported a Cu-catalyzed decarboxylative C(sp^3^)–N coupling employing NHPI esters as dual reagents [75] (Scheme 21A). The combination of CuCN and the ligands xantphos and neocuproine in a 2:3:1 ratio resulted in the formation of a light absorbing species with an absorption band in the 380–460 nm range. Thus, it was hypothesized that Cu^I^ catalytic species **109** would give rise to photoexcited complex **110** under blue light irradiation (Scheme 21B). Complex **110** and NHPI ester **3** would then engage in SET likely through an inner sphere mechanism, providing phthalimide ligated Cu^II^ intermediate **111** and carboxyl radical **112**. At this stage, the resulting radical species **9** would recombine with **111** to form Cu^III^ complex **113**. Alternatively, reaction of **3** with the photoexcited Cu^I^ complex through oxidative addition (OA) followed by decarboxylation could directly lead to intermediate **113** (Scheme 21B, blue arrow). However, radical clock experiments support the intermediacy of free radicals, indicating the involvement of the SET pathway (Scheme 21C). Eventually, reductive elimination of **113** afforded product **114** while regenerating the catalytic species **109**. It is worth nothing that further transformations of NHPI esters under photoinduced Cu catalysis have been reported in recent years, including decarboxylative alkynylations [76–78] and the C(sp^3^)–H alkylation of α-amino acids [79].

**Scheme 22:**
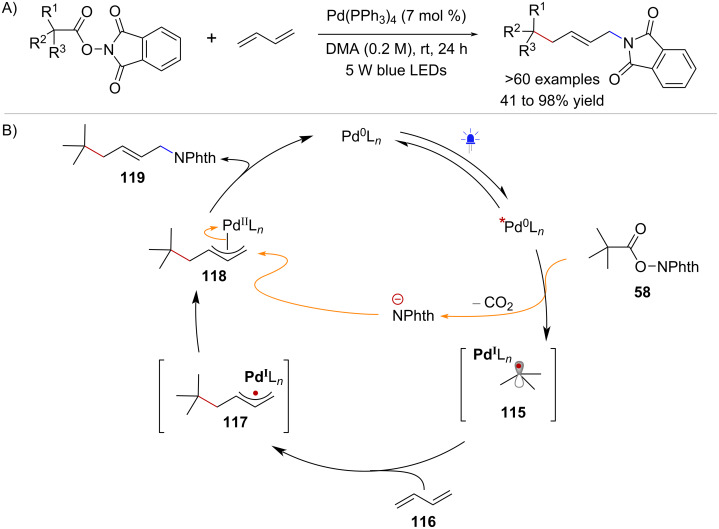
A) Photoinduced Pd-catalyzed aminoalkylation of 1,4-dienes. B) Proposed catalytic cycle.

The ability of NHPI esters to act as dual reagents was also investigated by Glorius and co-workers in the context of a photoinduced Pd-catalyzed aminoalkylation of 1,4-dienes [80] (Scheme 22A). The study showed that a photoexcited Pd^0^ species formed upon blue-light irradiation of Pd(Ph_3_)_4_, was able to promote the activation of NHPI esters via SET (Scheme 22B). The photoinduced electron transfer process between the Pd-photocatalyst and RAE **58** was proposed to form a hybrid alkyl Pd^I^-radical species **115** which could then react with 1,4-butadiene (**116**) to form hybrid allyl Pd^I^ complex **117**. Subsequently, it was suggested that the species **117** would lead to the formation of a π-allylpalladium intermediate **118** through radical recombination. Ultimately, nucleophilic attack by the phthalimidyl anion would generate the aminoalkylation product **119**, completing the catalytic cycle. In addition to this aminoalkylation method, the synthetic utility of radical intermediates derived from NHPI esters under photoinduced Pd-catalysis has been demonstrated in Heck-type couplings [81–82] and in the desaturation of aliphatic carboxylic acids [83].

### Initiation by metal catalysts and stoichiometric reductants

**Scheme 23:**
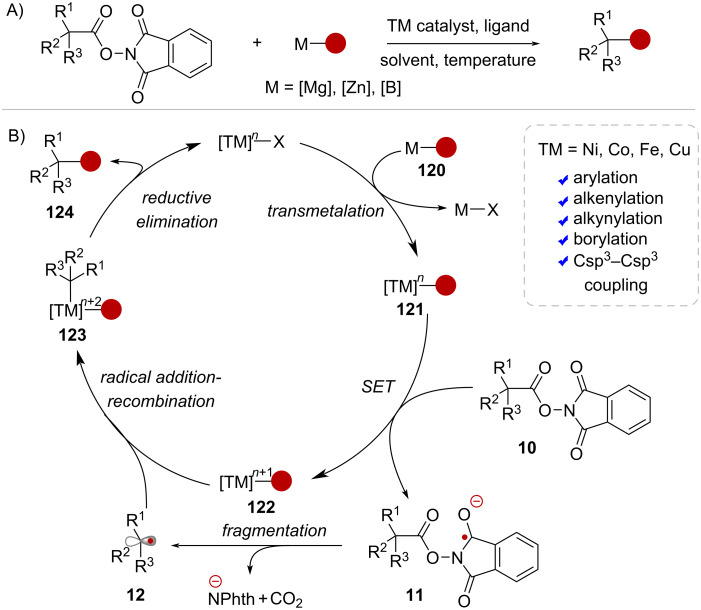
A) TM-catalyzed decarboxylative coupling of NHPI esters and organometallic reagents. B) Representative catalytic cycle.

The activation of NHPI esters under transition metal catalysis without the need of light is also feasible, and generally, two types of coupling reactions can be envisioned. On one hand, the decarboxylative cross-coupling (DCC) of NHPI esters with organometallic reagents, resembling classic Kumada, Negishi, and Suzuki couplings, has been enabled by nickel (Ni), cobalt (Co), iron (Fe), and copper (Cu) catalysts [84–91] (Scheme 23A). The typical mechanism begins by transmetallation of the organometallic coupling partner **120** to the TM catalyst (Scheme 23B). The resulting organometallic intermediate **121** can act as a reducing agent, transferring an electron to RAE **10** to form radical anion **11** and the corresponding oxidized metal complex **122**. Following fragmentation, the ensuing alkyl radical **12** is captured by intermediate **122**, resulting in the formation of complex **123**. At this point, the metal center has undergone a two-electron oxidation, making it well-suited for reductive elimination yielding the cross-coupling product **124**.

**Scheme 24:**
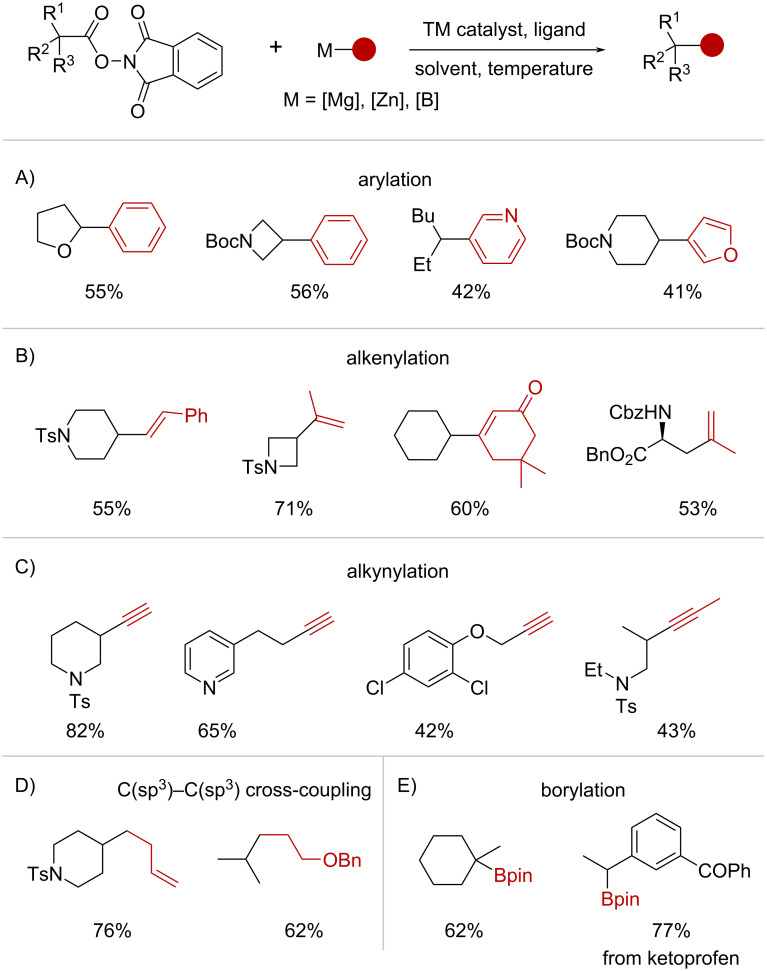
Synthetic applications of the TM-catalyzed decarboxylative coupling of NHPI esters and organometallic reagents.

Under these catalytic conditions, various TM-catalyzed decarboxylative functionalizations employing RAEs have been established (Scheme 24). Baran and co-workers have reported arylation protocols (Scheme 24A) using arylzinc reagents [84–85], Grignard reagents [85] and arylboronic acids [86], as well as decarboxylative alkenylation [87] (Scheme 24B), alkynylation [88] (Scheme 24C) and C(sp^3^)–C(sp^3^) cross-coupling [89] (Scheme 24D). Finally, similar chemistry has been extended to the decarboxylative borylation of RAEs under Ni [90] and Cu [91] catalysis (Scheme 24E). Importantly, the Wang group has independently studied the decarboxylative Negishi coupling of RAEs with organozinc reagents under Co-catalysis, effecting diverse arylation, alkenylation, and alkynylation reactions [92].

**Scheme 25:**
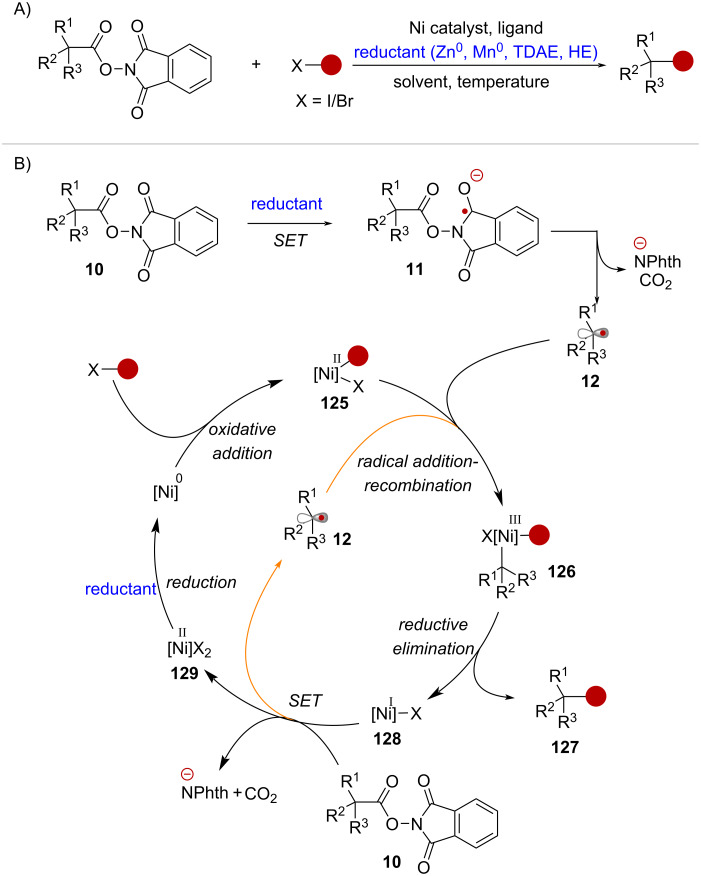
A) Ni-catalyzed cross-electrophile coupling of NHPI esters. B) Representative catalytic cycle.

The second type of reaction is referred to as cross-electrophile coupling and involves the Ni-catalyzed reaction of NHPI esters with aryl- and vinyl halides under reducing conditions (Scheme 25A). The general catalytic cycle begins with oxidative addition of an organohalide into a Ni^0^ catalyst yielding Ni^II^ complex **125** (Scheme 25B). In parallel, the initial SET activation of RAE **10**, leading to the formation of radical anion **11**, takes place in the presence of a stoichiometric reductant, which can be an organic reductant, such as tetrakis(*N*,*N*-dimethylamino)ethylene (TDAE) or Hantzsch ester (HE), or a metal such as zinc (Zn^0^) or manganese (Mn^0^). Upon fragmentation, radical species **12** is captured by the oxidative addition complex **125**, giving rise to Ni^III^ complex **126**. The cross-coupling product **127** is then formed via reductive elimination of **126** which gives Ni^I^ intermediate **128**. At this stage, it is proposed that the Ni^I^ complex **128** can participate in a SET event with another equivalent of substrate **10**, generating another equivalent of radical **12**, that propagates into the next catalytic cycle. Finally, the corresponding Ni^II^ complex **129** is reduced back to the corresponding Ni^0^ species by the stoichiometric reductant, initiating a new catalytic cycle.

**Scheme 26:**
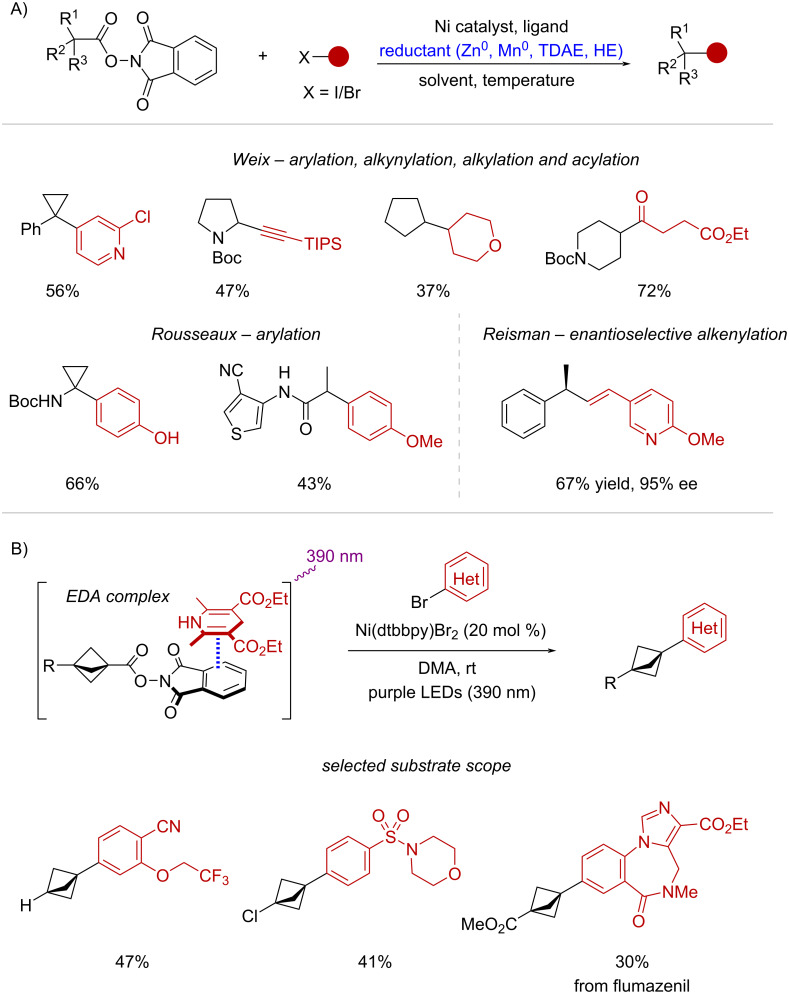
A) Synthetic applications of decarboxylative cross-electrophile couplings. B) Decarboxylative arylation of BCP-redox active esters enabled by EDA complex activation and Ni catalysis.

Weix and co-workers have developed a series of Ni-catalyzed couplings of NHPI esters with different electrophiles, including aryl halides [93–94], bromoalkynes [95], bromoalkanes [96], and pyridyl thioesters [97] (Scheme 26A). Likewise, the Rousseaux group has recently documented the arylation of NHPI esters obtained from cyclopropanecarboxylic acids [98] and malonic acid half amides [99], while the Reisman lab has pioneered an enantioselective cross-electrophile coupling between NHPI esters and alkenyl bromides [100] (Scheme 26A). In addition, Jolit and Molander disclosed the decarboxylative arylation of NHPI esters derived from bicyclo[1.1.1]pentanes (BCPs) by combining Ni-catalysis and photoinduced EDA complex activation [101] (Scheme 26B).

**Scheme 27:**
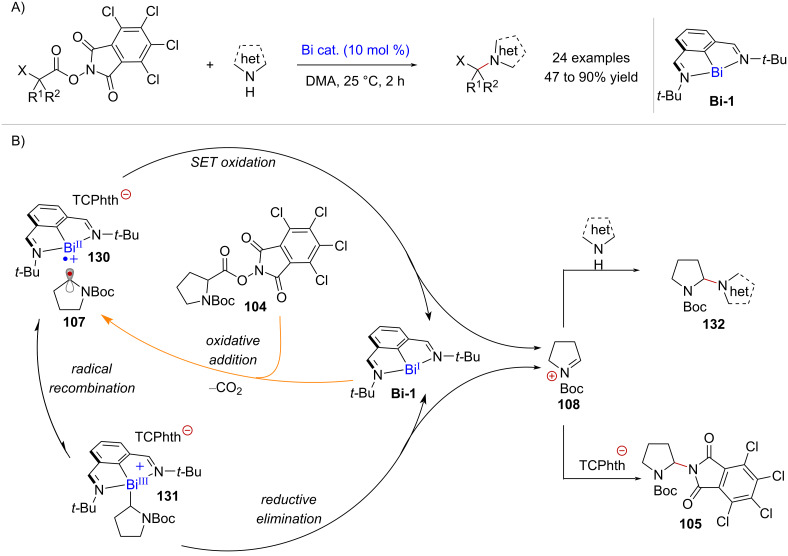
A) Activation of tetrachlorophthalimide redox-active esters enabled by a low-valency Bi complex. B) Proposed mechanism involving two plausible reaction pathways.

A novel approach for the activation of redox-active esters was recently reported by Cornella and co-workers [32]. In this study, low valent bismuth (Bi) complex **Bi-1** was found to exhibit redox properties similar to those of first row-transition metal catalysts, enabling the activation of tetrachlorophthalimide (TCPhth) active esters towards C(sp^3^)–N cross-couplings with nitrogen heterocycles (Scheme 27). The catalytic reaction was proposed to begin by oxidative addition of RAE **104** to catalyst **Bi-1**, forming an in cage radical pair consisting of Bi^II^ species **130** and α-amino radical **107** (Scheme 27B). Importantly, electron paramagnetic resonance (EPR) spectroscopy at 25 °C supported the formation of species **107**. However, NMR measurements at −40 °C showed the accumulation of Bi^III^ complex **131**, which can be prepared separately in a stoichiometric experiment. As such, two different pathways may lead to the C(sp^3^)–N cross-coupling products. On one hand, in-cage electron transfer from radical **107** to Bi^II^ complex **130** can generate iminium ion **108**. Alternatively, intermediate **108** could arise from complex **131** by reductive elimination. Ultimately, the iminium ion can be trapped by the nitrogen nucleophile to form product **132** or by the in-situ-generated tetrachlorophthalimyl anion (^–^TCPhth) affording product **105**.

**Scheme 28:**
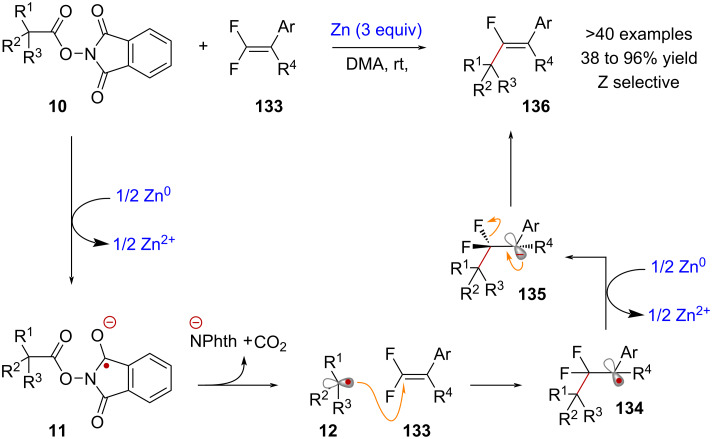
Activation of NHPI esters mediated by Zn^0^ applied in a *Z*-selective alkenylation reaction.

Stoichiometric reductants can facilitate certain radical-mediated transformations that involve NHPI esters, even without the presence of metal catalysts. Larionov and Sun have independently reported notable examples of these transformations, involving the Zn^0^-mediated cross-coupling of redox-active esters with chlorophosphines [102] and *gem*-difluoroalkenes [103], respectively. In the report by Sun and co-workers, it was proposed that the activation of RAE **10** occurs upon reduction with Zn powder to give radical anion **11** (Scheme 28). Following fragmentation, radical intermediate **12** would then attack *gem*-difluoroalkene **133**, affording intermediate **134**. Reduction of this radical species by another equivalent of Zn^0^ would then form anionic intermediate **135**. Finally, the selective formation of *Z*-monofluoroalkene product **136** is achieved through anticoplanar elimination of fluoride.

**Scheme 29:**
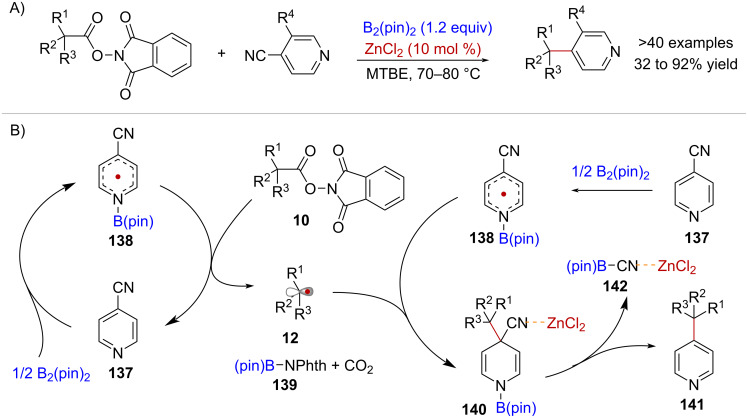
A) Activation of NHPI esters enabled by a pyridine-boryl radical species applied to the decarboxylative alkylation of pyridines. B) Reaction mechanism.

Shuhua Li and co-workers reported the generation of alkyl radicals from NHPI esters, mediated by a pyridine-boryl radical reductant species in the context of alkene hydroalkylation [104] and cross-decarboxylative couplings with 4-cyanopyridines [105] (Scheme 29A). In the mechanism of the latter transformation, pyridine **137** also serves as a catalyst, generating the crucial pyridine-boryl radical **138** via reaction with B_2_pin_2_ (Scheme 29B). The proposed species **138** induces the reductive fragmentation of active ester **10**, regenerating pyridine **137** while forming alkyl radical **12**, CO_2_ and phthalimide–B(pin) adduct **139**. Subsequently, radical–radical coupling between **12** and one equivalent of **138** affords dihydropyridine **140**, which upon re-aromatization, facilitated by ZnCl_2_ acting as a Lewis acid, yields product **141**, accompanied by the formation of cyano-B(pin) **142**. Importantly, in the absence of ZnCl_2_, intermediate **140** could be detected by HRMS and ^11^B NMR, highlighting the pivotal role of ZnCl_2_ in promoting rearomatization.

### *N*-Heterocyclic carbene (NHC) catalysis

**Scheme 30:**
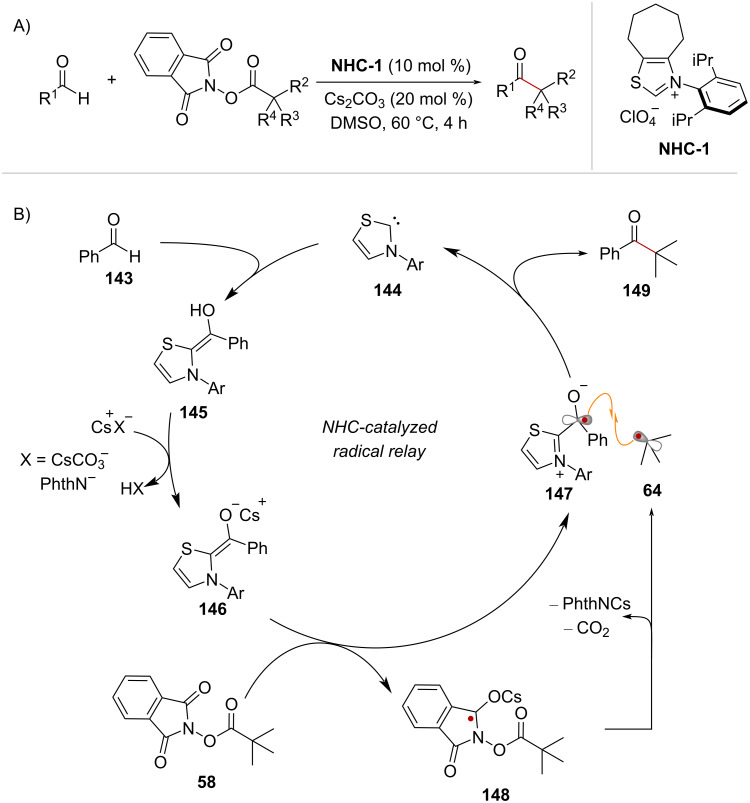
A) Decarboxylative coupling of RAE and aldehydes enabled by NHC-catalyzed radical relay. B) Proposed reaction mechanism.

In 2019, Nagao and Ohmiya reported the decarboxylative coupling of NHPI esters with aldehydes enabled by NHC catalysis [106]. The reaction proceeds with the catalyst **NHC-1** at 60 °C and gives rise to ketones (Scheme 30A). The proposed mechanism begins with the reaction of benzaldehyde (**143**) and the NHC-catalyst **144** to form the neutral Breslow intermediate **145** (Scheme 30B). Then, cesium carbonate deprotonates the enol OH in **145**, to provide the enolate form of Breslow’s intermediate **146**, which is a suitable reducing agent to trigger the fragmentation of NHPI ester **58** (*E*°_ox_ = −0.97 V vs SCE in MeCN). Hence, it is proposed that enolate **146** induces the single electron reduction of **58**, generating the persistent radical **147** and the transient species **148**, which fragments into *tert*-butyl radical (**64**). Notably, the reduction of NHPI ester **58** could be facilitated by interaction of a cesium cation with the oxygen lone pair of the phthalimide, in analogy to the mechanism discussed in Scheme 7B. Lastly, radical–radical coupling between **64** and **147**, accompanied by elimination of the NHC catalyst, yields ketone product **149**. In subsequent studies, this NHC-catalyzed radical relay activation mode has been extended to the alkylation of aliphatic aldehydes [107] and to the three-component alkylacylation of olefins [108].

**Scheme 31:**
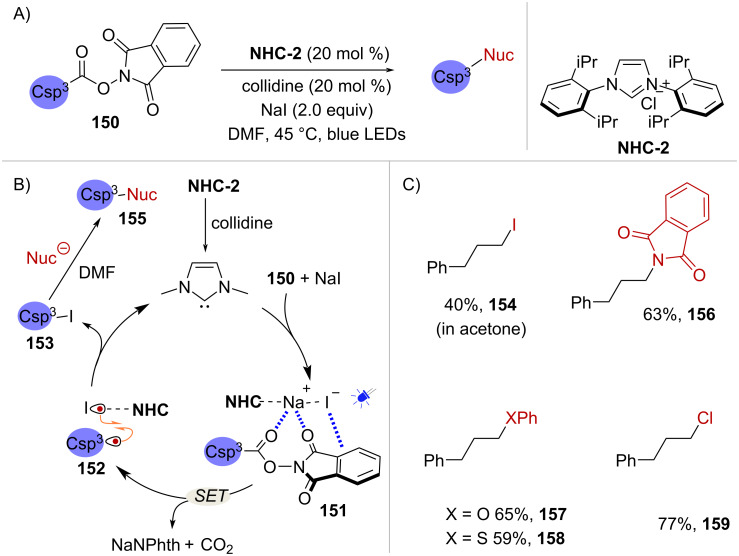
A) Decarboxylative C(sp^3^)–heteroatom coupling reaction of NHPI esters under NHC catalysis B) The NHC catalyst improves reaction efficiency by EDA complex stabilization. C) Examples of substrate scope.

Alternatively, NHC catalysts can mediate the generation of radical intermediates from NHPI esters via the stabilization of a photoactive EDA complex. In 2020, Wang and Chen reported a photochemical C(sp^3^)-heteroatom coupling reaction of NHPI esters mediated by NaI and the catalyst **NHC-2** [109] (Scheme 31A). Initial investigations showed that the reaction proceeded in the absence of the NHC catalyst, and it was reasoned that substrate **150** and NaI formed EDA complex **151** that would undergo light-mediated decarboxylation (Scheme 31B). However, upon addition of catalyst **NHC-2** an improvement of the reaction yield from 36% to 63% was observed. The authors proposed that the addition of the NHC catalyst facilitates the formation of EDA complex **151**. In addition, it is thought that the stabilization imparted by the NHC catalyst is crucial in the subsequent radical recombination between C(sp^3^) radical **152** and the corresponding iodine-centered radical which provides iodination product **153** (Scheme 31B). It is worth noting that the iodination product is formed exclusively when using acetone as the solvent (see compound **154** in scheme C) whereas in DMF a nucleophilic substitution takes place to afford products of the type **155** (Scheme 31B). Representative examples of the substrate scope are shown in Scheme 31C. The in situ-generated phthalimidyl anion (–Nphth) is a competent nucleophile and gives rise to primary protected amines such as **156**. Additionally, sodium phenolate and thiophenolate salts give rise to products **157** and **158**, respectively, while the chlorination product **159** was obtained upon the addition of tetrabutylammonium chloride (Scheme 31C).

### Mechanisms under electrochemical activation

The electrochemical activation of NHPI esters provides significant advantages compared to other methods. It allows for single electron reduction to be facilitated by cost-effective carbon-based cathodes, eliminating the requirement for precious metal photocatalysts or exogeneous reductants such as Zn^0^. In the final section of this perspective, we explore examples where NHPI esters have been utilized as radical precursors under electrochemical conditions.

**Scheme 32:**
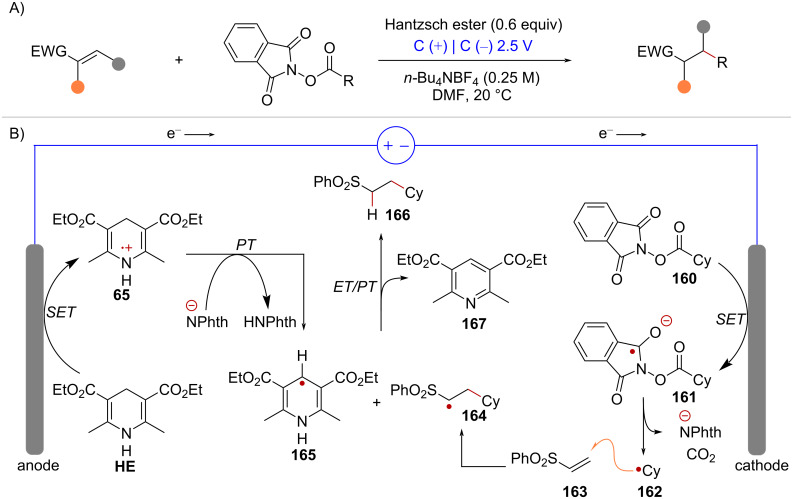
A) Electrochemical Giese-type radical addition of NHPI esters. B) Reaction mechanism.

Giese-type radical additions, which are usually performed under conditions of photoredox-catalysis (see Scheme 4), can also be achieved under constant-potential electrolysis employing graphite electrodes [110] (Scheme 32A). Under these conditions, activation of NHPI ester **160** by SET occurs at the cathode’s surface affording the corresponding radical anion **161** (Scheme 32B). Subsequent fragmentation leads to the cyclohexyl radical (**162**) which then adds to the terminal carbon of radical acceptor **163**, leading to radical intermediate **164**. In the other redox half-reaction, Hantzsch ester (**HE**) undergoes anodic oxidation to form radical cation **65**, which then transfers a proton, likely to the phthalimidyl anion (**^–^**Nphth), resulting in the formation of radical species **165**. Finally, reaction between intermediates **164** and **165** through sequential electron transfer and proton transfer (ET/PT) leads to the hydroalkylation product **166** and the pyridine byproduct **167**.

**Scheme 33:**
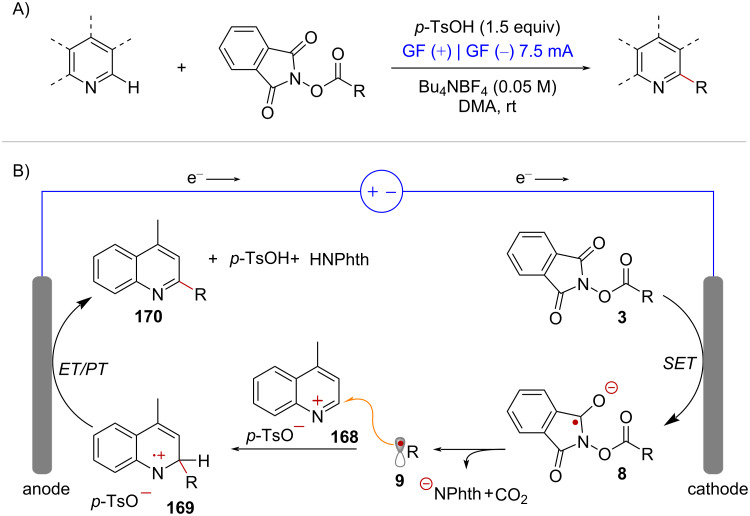
Electrochemical Minisci-type radical addition of NHPI-esters.

In addition, there have been reports of Minisci-type additions where radical intermediates are electrochemically generated from NHPI esters [111–113] (Scheme 33A). The mechanism of this redox neutral reaction involves reductive fragmentation of the radical precursor **3** mediated by the cathode under constant-current electrolysis (Scheme 33B). The resulting alkyl radical **9** attacks the protonated quinoline **168**, forming radical cation intermediate **169**. Finally, single electron oxidation of **169** at the anode, followed by rearomatization via proton-transfer forms the alkylated heterocycle **170**.

**Scheme 34:**
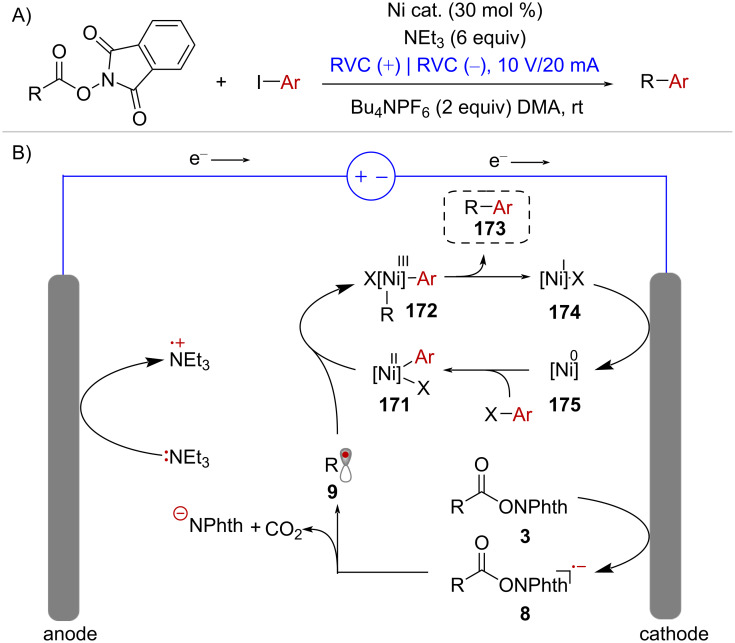
Ni-electrocatalytic cross-electrophile coupling of NHPI esters with aryl iodides.

As discussed in Scheme 25, the Ni-catalyzed cross-electrophile coupling between redox-active esters and aryl halides requires the addition of a stoichiometric reductant (typically Zn^0^ or Mn^0^) to both activate the NHPI ester and turn-over the catalytic cycle. However, the merger of Ni-catalysis and electrochemistry allows for the implementation of more convenient conditions in which these two crucial reductive steps can be mediated by the cathode (Scheme 34). In this context, Jamison and co-workers developed an electrochemically driven decarboxylative C(sp^3^)–C(sp^2^) cross-coupling protocol that proceeds in a divided electrochemical cell with reticulated vitreous carbon (RVC) electrodes and triethylamine (NEt_3_) as sacrificial reductant [114] (Scheme 34A). In the proposed mechanism, radical species **9**, which is generated upon cathodic reduction of active ester **3**, is captured by complex **171** (Scheme 34B). The resulting Ni^III^ complex **172** undergoes facile reductive elimination to form cross-coupling product **173** and Ni^I^ intermediate **174**. Finally, reduction of **174** at the cathode surface restores the Ni^0^ species **175** giving rise to a new catalytic cycle.

**Scheme 35:**
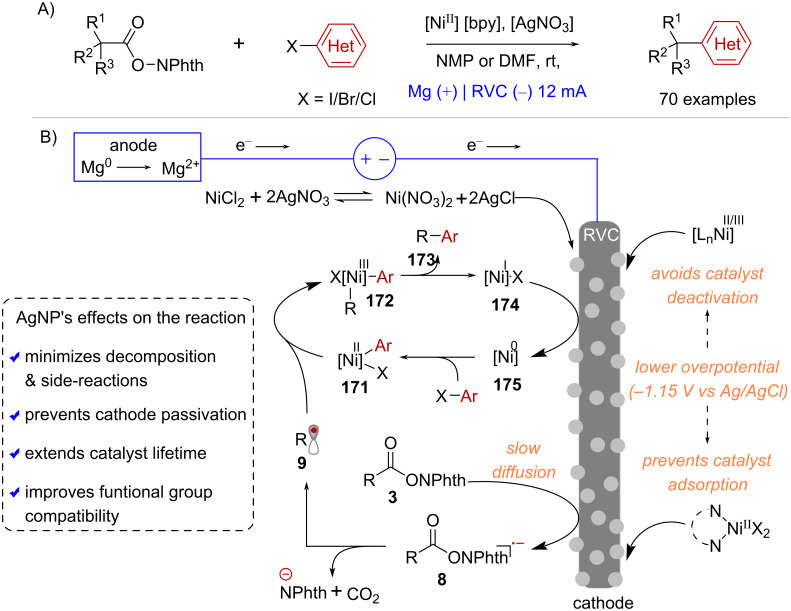
A) Decarboxylative arylation of NHPI esters under Ag-Ni electrocatalysis B) Formation of AgNP on the cathode’s surface and its effect on the reaction.

Reductive cross-electrophile couplings that incorporate redox-active esters and aryl halides have the potential to simplify the syntheses of drug-like compounds through C(sp^3^)–C(sp^2^) bond formation. However, their synthetic utility is frequently restricted due to various challenges, such as RAE decomposition and a limited aryl halide scope. In recent years, the Baran lab has made progress in enhancing the practicality and applicability of electrochemically driven decarboxylative couplings involving NHPI esters and aryl-halides [115]. Their optimized reaction conditions required a Ni^II^ precursor, 2,2’-bipyridine (bpy) as ligand, silver nitrate (AgNO_3_) as an additive and the combination of a magnesium (Mg) sacrificial anode and a RVC cathode (Scheme 35A). A crucial discovery in advancing this methodology was the in situ formation of silver nanoparticles (AgNP) on the cathode's surface [116] (Scheme 35B). The use of this Ag-doped cathode led to slower mass transport and minimized side reactions caused by rapid reduction of RAEs, thereby avoiding substrate decomposition and enhancing reaction yields (Scheme 35B). Furthermore, the presence of the AgNP layer on the cathode caused a decrease of the reaction’s overpotential from –1.66 V to –1.15 V (vs Ag/AgCl), which is thought to prevent catalyst deactivation via successive reduction of the various Ni species, while also avoiding electrode passivation caused by catalyst adsorption (Scheme 35B).

**Scheme 36:**
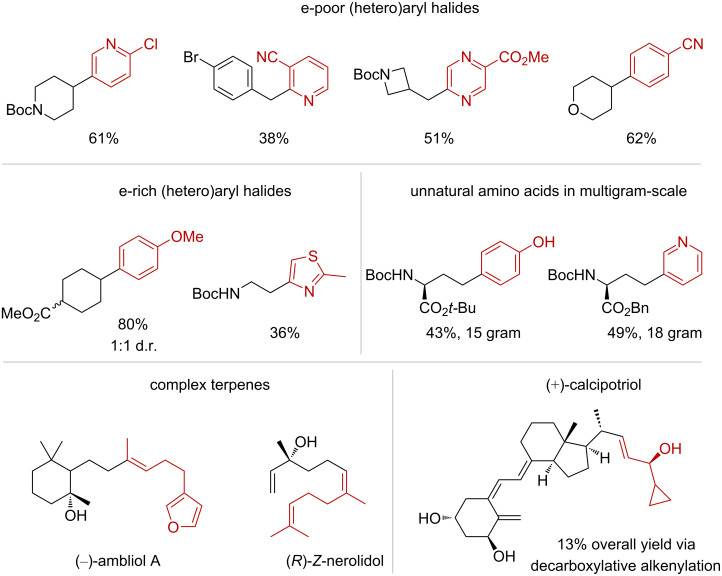
Synthetic applications of decarboxylative couplings of NHPI esters under Ni-electrocatalysis.

Baran and co-workers have demonstrated that these overall milder reaction conditions greatly improved the functional group compatibility of decarboxylative couplings under reducing conditions. For example, the cross-coupling between NHPI esters and both electron-poor and electron-rich (hetero)aryl halides was equally effective (Scheme 36). In addition, the preparation of unnatural amino acids in multigram scale [115], along with the syntheses of complex terpenes [116] and (+)-calcipotriol [117] have showcased the vast synthetic potential and broad applicability of NHPI esters activated under these electrochemical conditions (Scheme 36).

## Conclusion

Given their rich history and their continued use in diverse methodologies, *N*-hydroxyphthalimide esters have been and will remain to be important tools in synthetic organic chemistry. In this perspective, we have surveyed recent advancements in photochemistry, TM catalysis, NHC catalysis, and electrochemistry to show the generality of these RAEs in diverse mechanistic paradigms. Their application as radical progenitors continues to broaden the scope of radical-mediated reactions, especially in complex molecular settings, where issues of chemoselectivity can benefit from the multitude of mechanisms for their activation.

**Scheme 37:**
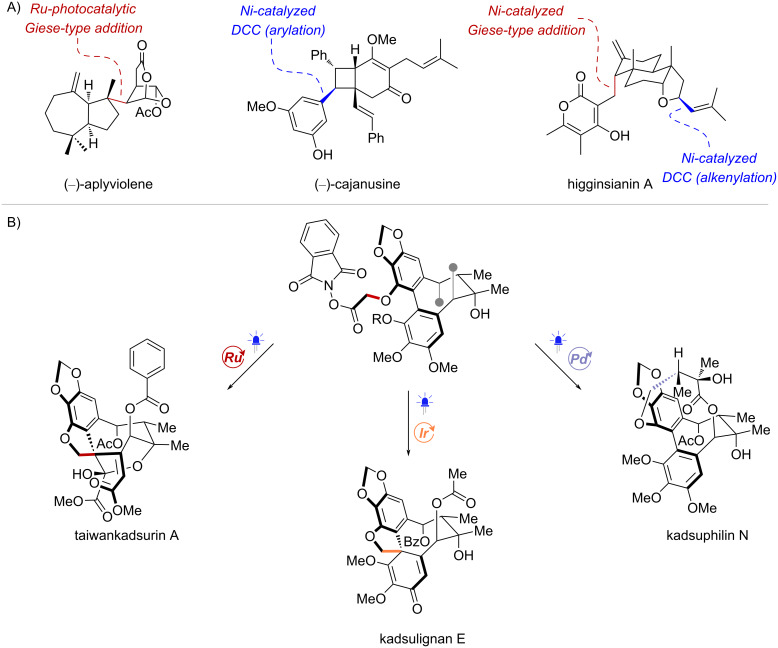
Examples of natural product syntheses in which RAEs were used in key C–C bond forming reactions.

Particularly, the use of RAEs in natural product total synthesis enables the assembly of strategic C–C bonds through radical coupling reactions that can draw from a wide range of reaction conditions (Scheme 37). Pioneering work by Schnermann and Overman demonstrated the early potential of photocatalytic Giese-type additions, successfully coupling two complex ring fragments and creating adjacent stereocenters, which effectively solved major challenges in the synthesis of (–)-aplyviolene [38] (Scheme 37A). Brown and co-workers devised a diastereoselective Ni-catalyzed decarboxylative arylation as a crucial step in the synthesis of (–)-cajanusine [118] (Scheme 37A). Likewise, the Baran lab employed Ni-catalyzed decarboxylative couplings of RAEs to form two strategic C–C bonds in the synthesis of higginsianin A [119] (Scheme 37A). Recently, Lumb and co-workers showcased the use of NHPI esters in key radical cyclizations, allowing the synthesis of diverse dibenzocyclooctadiene lignans [55] (Scheme 37B). A Ru-catalyzed cyclization was employed to construct the quaternary stereocenter in taiwankadsurin A, whereas the corresponding spirocycle in kadsulignan E was formed under Ir photocatalysis. In addition, photoinduced Pd catalysis was applied in the key macrocyclization step to complete the synthesis of kadsuphilin N.

These applications in total synthesis serve as a testament to the synthetic versatility of radical reactions facilitated by the use of RAEs. In the years to come, we anticipate that the continued development of new catalysts and the implementation of increasingly mild reaction conditions will open new possibilities for further applications of NHPI esters in radical reactions. Expanding the mechanisms and reactivity discussed herein will continue to improve our understanding of radical chemistry toward exploring novel chemical space.
